# Finding a partner in the ocean: molecular and evolutionary bases of the response to sexual cues in a planktonic diatom

**DOI:** 10.1111/nph.14557

**Published:** 2017-04-21

**Authors:** Swaraj Basu, Shrikant Patil, Daniel Mapleson, Monia Teresa Russo, Laura Vitale, Cristina Fevola, Florian Maumus, Raffaella Casotti, Thomas Mock, Mario Caccamo, Marina Montresor, Remo Sanges, Maria Immacolata Ferrante

**Affiliations:** ^1^ Integrative Marine EcologyStazione Zoologica Anton DohrnVilla Comunale 1Naples80121Italy; ^2^ Earlham InstituteNorwich Research ParkNorwichNR4 7UGUK; ^3^ URGIINRAUniversité Paris‐Saclay78026VersaillesFrance; ^4^ School of Environmental SciencesUniversity of East AngliaNorwich Research ParkNorwichNR4 7TJUK; ^5^ Biology and Evolution of Marine OrganismsStazione Zoologica Anton DohrnVilla Comunale 1Naples80121Italy

**Keywords:** algae, diatom, genomics, mating type, phytoplankton, *Pseudo‐nitzschia multistriata*, sexual reproduction, signal transduction

## Abstract

- Microalgae play a major role as primary producers in aquatic ecosystems. Cell signalling regulates their interactions with the environment and other organisms, yet this process in phytoplankton is poorly defined. Using the marine planktonic diatom *Pseudo‐nitzschia multistriata*, we investigated the cell response to cues released during sexual reproduction, an event that demands strong regulatory mechanisms and impacts on population dynamics.
- We sequenced the genome of *P. multistriata* and performed phylogenomic and transcriptomic analyses, which allowed the definition of gene gains and losses, horizontal gene transfers, conservation and evolutionary rate of sex‐related genes. We also identified a small number of conserved noncoding elements.
- Sexual reproduction impacted on cell cycle progression and induced an asymmetric response of the opposite mating types. G protein‐coupled receptors and cyclic guanosine monophosphate (cGMP) are implicated in the response to sexual cues, which overall entails a modulation of cell cycle, meiosis‐related and nutrient transporter genes, suggesting a fine control of nutrient uptake even under nutrient‐replete conditions.
- The controllable life cycle and the genome sequence of *P. multistriata* allow the reconstruction of changes occurring in diatoms in a key phase of their life cycle, providing hints on the evolution and putative function of their genes and empowering studies on sexual reproduction.

## Introduction

Phytoplankton feature prominently in aquatic ecosystems, showing striking morphological and functional diversity and accounting for one‐half of the Earth's primary productivity (Falkowski & Knoll, 2011). Diatoms are a major component of phytoplankton with over 100 000 species (Mann & Vanormelingen, 2013) and contribute substantially to primary production and major biogeochemical cycles (Armbrust, 2009). A high rate of DNA turnover, horizontal gene transfer (HGT) from bacteria and endosymbiotic events are responsible for the chimeric nature of diatom genomes, which have probably contributed to the heterogeneity of their physiological and ecological traits (Bowler *et al*., 2010).

The first assembled genomes of a centric (*Thalassiosira pseudonana*, Armbrust *et al*., 2004) and a pennate (*Phaeodactylum tricornutum*, Bowler *et al*., 2008) diatom were small in size (27–32 Mb) with 10 000–14 000 genes. They contained only one‐half of the genes with an annotated function, and *c*. 35% of the genes were reported to be species specific. Further, *c*. 5% of *P. tricornutum* genes were predicted to be acquired by HGT from bacteria. These genomes contributed towards an understanding of the genes and pathways involved in nutrient assimilation and metabolism of diatoms. To improve our understanding of the evolution and adaptation of this highly diverse group of organisms, additional diatom genomes were sequenced, such as those of the open‐ocean centric diatom *Thalassiosira oceanica* (Lommer *et al*., 2012), the oleaginous *Fistulifera solaris* (Tanaka *et al*., 2015) and the polar diatom *Fragilariopsis cylindrus* (Mock *et al*., 2017), instrumental for the study of iron physiology, lipid metabolism and adaptation to cold, respectively.

The dynamics of planktonic communities are strongly dependent on the life cycle traits of the individual species. Diatoms have a unique life cycle characterized by progressive cell size reduction in the population, imposed by a rigid silica wall. A few exceptions apart, sexual reproduction is an obligate phase in diatom life cycles, important not only to generate genetic diversity, but also to escape the miniaturization process, thus allowing the persistence of populations by restoring the original cell size (Montresor *et al*., 2016). It has been proposed that some of the unique features of the diatom genomes may reflect the unusual characteristics of diatom life cycles (Bowler *et al*., 2008). However, the most widely used diatom models are putatively asexual and this has hampered research on the molecular and genomic underpinning of sexual reproduction.

**Figure 1:**
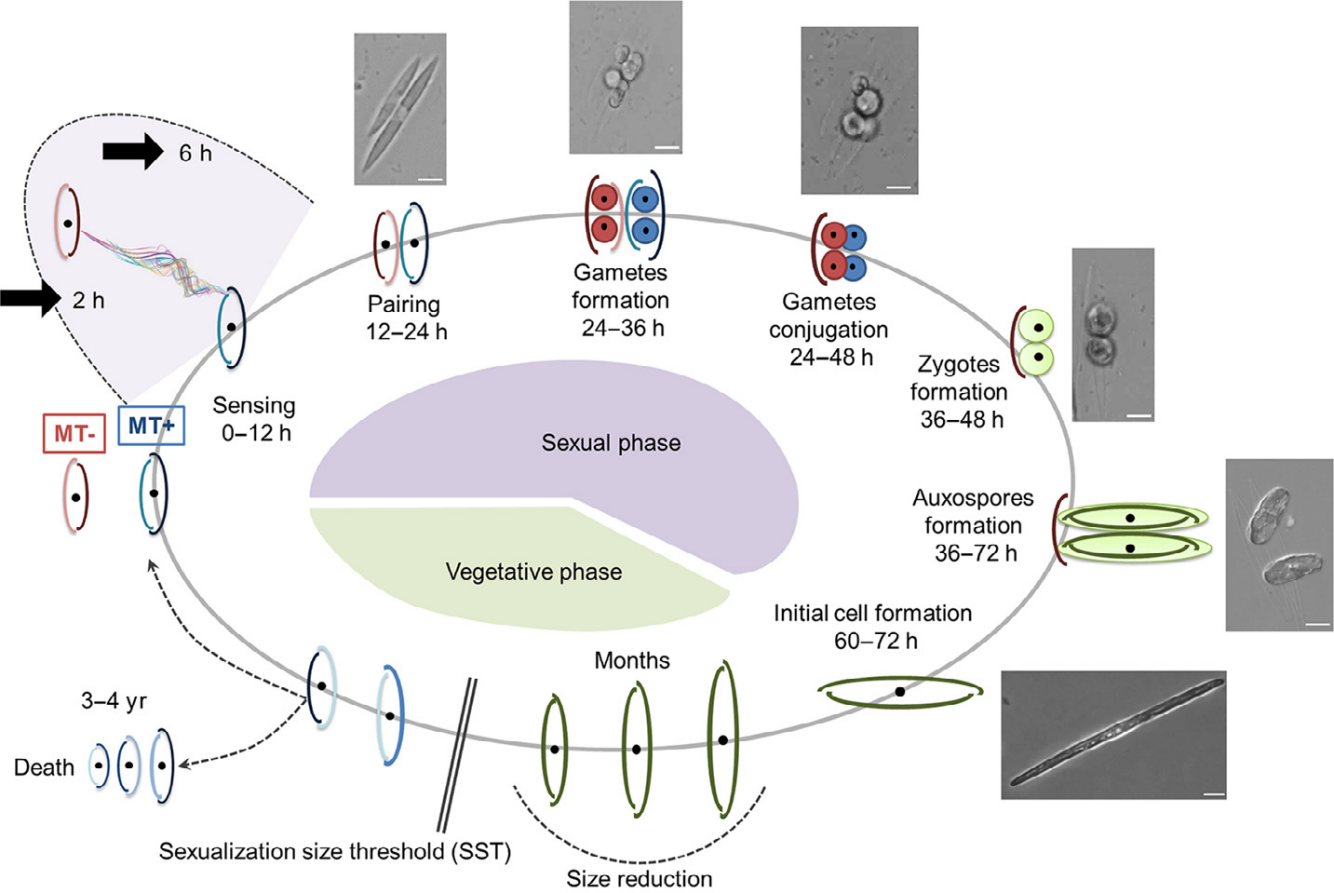
Schematic drawing of the life cycle of *Pseudo‐nitzschia multistriata*. Starting clockwise from the bottom portion of the cycle, the vegetative phase is characterized by progressive cell size reduction of the population imposed by the rigid silica wall, made up of two unequal thecae. During this process, the cells reach the sexualization size threshold (SST) and can either keep decreasing in size until death, or undergo sexual reproduction and escape the miniaturization process, producing large cells. In the heterothallic *P. multistriata*, sex can occur only if strains of opposite mating type come into contact. The perception of chemical cues deriving from the mating partner (0–12 h) brings cells of opposite mating type to pair (12–24 h). The formation of gametes (24–36 h) takes place following meiosis. Conjugation of the haploid gametes (24–48 h) produces two expandable zygotes (36–48 h) that develop into auxospores (36–72 h). Within each auxospore, an initial cell of maximum size is synthesized (60–72 h), restoring the vegetative phase of the cycle. The time interval for each stage is indicated. Representative microscopic images of the different stages are shown outside the circle; bar, 10 μm. Thick black arrows mark the sampling time points for the experiments described in this work. MT, mating type.

The marine planktonic pennate diatom *Pseudo‐nitzschia multistriata* has a typical, controllable size reduction–restitution life cycle in which cells of opposite mating type (MT+ and MT−) produce gametes when they are below the size threshold for sex (D'Alelio *et al*., 2009). On gamete conjugation, an expandable zygote is produced, within which the cell of maximum size is formed (Fig. 1). Sexual reproduction requires a threshold cell concentration to start, suggesting that chemical signalling is needed to allow the induction of the sexual phase in this species (Scalco *et al*., 2014). Diffusible chemical cues have been shown to be responsible for a multi‐step sexualization process, and two sex pheromones have been characterized for the benthic diatom *Seminavis robusta* (Gillard *et al*., 2013; Moeys *et al*., 2016). The availability of transcriptomic data for the latter species and for *P. multistriata*, coupled with a comparative genomic approach, led us to the identification of the diatom genes involved in meiosis (Patil *et al*., 2015). Although the meiosis toolkit is well conserved, the sexual cues and the response mechanisms might have diverged substantially between benthic species, which can glide on the substrate to follow attraction cues, and planktonic species, which are suspended in the water column. Indeed, it is a mystery how pennate planktonic diatoms find their partner and the significance of pheromone signalling remains completely unexplored.

*Pseudo‐nitzschia multistriata* is able to produce the neurotoxin domoic acid, a molecule that can contaminate seafood and cause a syndrome called amnesic shellfish poisoning (Trainer *et al*., 2012). The genome sequence of this toxic species and insights into the mechanisms underlying its life cycle regulation will facilitate investigations on the dynamics of toxic *Pseudo‐nitzschia* blooms.

We chose *P. multistriata* as a model to study sexual reproduction. We report the assembly and annotation of its genome, which was first exploited to reveal unexplored features of diatom genomes, such as conserved noncoding elements (CNEs) with a potential regulatory function, and transposable element activity. We also assessed the turnover of gene families amongst Stramenopiles through an in‐depth phylogenomic approach to better identify conserved and unique features of *P. multistriata*, and to provide novel information on HGT. Furthermore, the availability of the *P. multistriata* genome, coupled with a transcriptomic approach, led us to dissect the signalling pathways employed in the early phases of sexual reproduction. Several mating type (MT)‐specific gene expression changes were observed, highlighting the involvement of different pathways in the response to putative pheromones, whereas other changes were common to both MTs, including growth arrest and the modulation of cell cycle genes and nutrient transporters.

## Materials and Methods

### Strains

**Figure 2:**
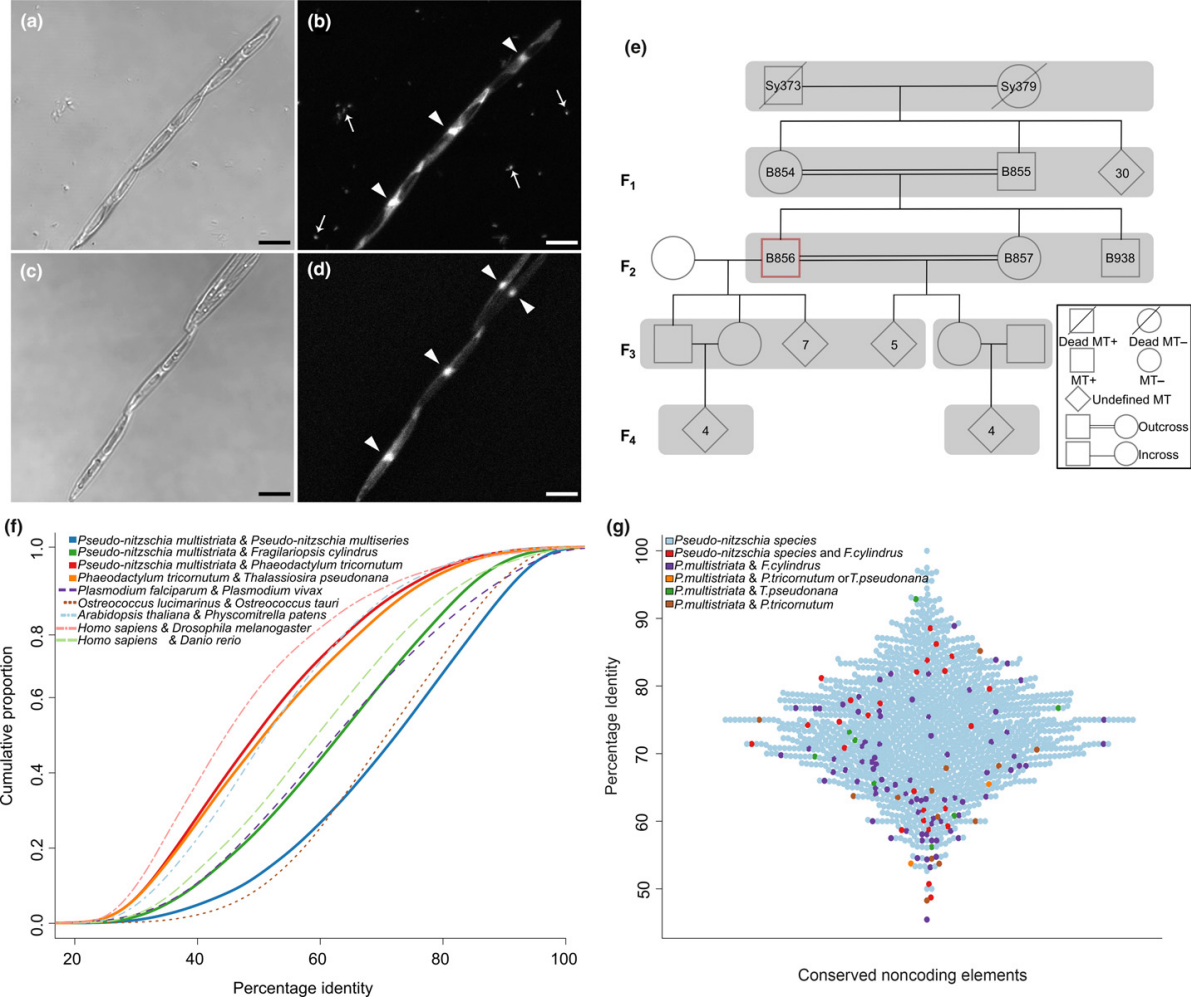
Main features of *Pseudo‐nitzschia multistriata* and its genome. (a, b) Microscopic images showing three cells in a chain in a normal culture with bacteria, in bright field and fluorescence, respectively, and (c, d) four cells in an axenic culture without bacteria. DAPI (4′,6‐diamidino‐2‐phenylindole) stains DNA in cell nuclei (arrowheads) and bacterial nucleoids (thin arrows). Bars, 10 μm. (e) *Pseudo‐nitzschia multistriata* pedigree showing four generations. Strain B856 was used to produce the genome sequence. (f) Estimation of species divergence based on amino acid identity of coding genes. The *x*‐axis represents the average percentage identity of BLASTp hits with maximum scores for the first species against the second. The *y*‐axis represents the cumulative proportion of the genes showing a given percentage identity. (g) Distribution of percentage identity for noncoding elements conserved between *Pseudo‐nitzschia* species (light blue dots), among *P. multistriata*,* Pseudo‐nitzschia multiseries* and *Fragilariopsis cylindrus* (red dots) and in other combinations. The *x*‐axis represents the identified conserved noncoding elements, stacked for best visualization of their distribution of conservation.

A *Pseudo‐nitzschia multistriata* (Takano) Takano pedigree was built starting from two strains collected in 2009 (Fig. 2). Strain B856, chosen for genome sequencing, was made axenic by treatment with antibiotics (Supporting Information Methods S1). RNA‐seq reads used to produce the *de novo* transcriptome were obtained from strains Sy373, Sy379, B856 and B857 (Fig. 2e). For the differential expression studies, strains B856, B857 and B938 were used with B936, B937 and B939, isolated from the LTER (Long TERm) station MareChiara (40°48.5′N, 14°15′E). Cultures were kept at a temperature of 18°C, irradiance of 80 μmol photons m^−1^ s^−1^ and in a 12 h : 12 h light : dark photoperiod.

### Genome sequencing and assembly

B856 cells were collected onto 1.2‐μm pore‐size membrane filters (RAWP04700 Millipore) and DNA was extracted with phenol–chloroform as described in Sabatino *et al*. (2015). The *P. multistriata* genome was assembled from a total of 172 million 101‐bp overlapping paired‐end reads with *c*. 175‐bp inserts, 117 million 100‐bp paired‐end reads with *c*. 450‐bp inserts, 72 million *c*. 68‐bp (after trimming) mate pair reads with *c*. 1.2‐kb inserts and 5.4 million *c*. 156‐bp (after trimming) mate pair reads with *c*. 4.5‐kb inserts. Mate pair libraries were processed by NextClip to remove adapters. Depending on the library, the genome size was estimated to be between 71 and 82 Mb using SGA preqc. Reads from libraries exceeding 100× coverage were randomly subsampled to 100× and then assembled into scaffolds by Allpaths‐LG (Gnerre *et al*., 2011) via Rampart (Mapleson *et al*., 2015). The completeness of the genome was evaluated using Cegma with the set of 248 core eukaryotic genes (CEGs) (Parra *et al*., 2007). The assembly (accession number PRJEB9419) can be visualized at http://apollo.tgac.ac.uk/Pseudo-nitzschia_multistriata_V1_4_browser/sequences (username and password: pnitzschia).

### Gene prediction and annotation

Protein‐coding genes were predicted using a workflow incorporating RNA‐seq reads, homologous proteins from *P. tricornutum*,* T. pseudonana* and a *de novo P. multistriata* transcriptome assembly. RNA‐seq reads were combined from four different libraries (samples: B856, libraries HCUO and HCUH; B857, libraries HCUN and HATT; available at http://genomeportal.jgi.doe.gov/pages/dynamicOrganismDownload.jsf?organism=PsenittraphaseII) and assembled *de novo*. The transcripts generated were used as training data for Augustus (Stanke *et al*., 2006). The model built on the training data was applied to the entire repeat masked assembly, together with external support from homologous proteins aligned using Exonerate (Slater & Birney, 2005). The predicted gene models were annotated using Annocript (Musacchia *et al*., 2015).

Repeats were identified using Repet. The TEdenovo pipeline (Flutre *et al*., 2011) was used to build a library of consensus sequences of repetitive elements in the genome assembly. The TEannot pipeline (Quesneville *et al*., 2005) was employed with default settings using the sequences from the filtered combined library as probes to perform genome annotation.

Full‐length complete long terminal repeats (LTRs) were identified using LTRHarvest and LTRDigest (Gremme *et al*., 2013). The relative age of LTR insertion was estimated using the method proposed in previous studies (Kimura, 1980).

The statistics for the genomic features in Table 1 were extracted from the GFF files using shell scripts and the BEDTools package (Quinlan, 2014). The genome size, N50 value and GC content were taken from the respective publications (Armbrust *et al*., 2004; Bowler *et al*., 2008; Cock *et al*., 2010; Lévesque *et al*., 2010; Lommer *et al*., 2012; Tanaka *et al*., 2015; Mock *et al*., 2017).

**Table 1:** Genome assembly and gene annotation statistics for selected Stramenopiles

Organism	*Pseudo‐nitzschia multistriata*	*Pseudo‐nitzschia multiseries*	*Fragilariopsis cylindrus*	*Fistulifera solaris*	*Phaeodactylum tricornutum*	*Thalassiosira pseudonana*	*Thalassiosira oceanica*	*Pythium ultimum*	*Ectocarpus siliculosus*
Genome size (Mb)	59	219	61	50	27	32	92	45	196
N50 (Mb)	0.138	0.147	1.3	na	0.945	1.9	0.04	0.837	0.504
G + C (%)	46.3	40.72	39.8	46.1	48.8	47	53.3	52	53.6
Repeat (%)	25	73	38	16	6.4	2	na	7	22.7
Gene count	12 008	19 703	21 066	20 621	10 402	11 776	34 500	15 291	16 256
Av. gene length (bp)	2205	1522	1575	na	1511	1553	1256	1312	1526
Av. exon length (bp)	1164	509	625	na	842	612	464	501	242
Av. intron length (bp)	341	229	245	na	135	124	149	116	715
Av. exon number per gene	1.87	2.38	2.08	na	1.79	2.49	2.3	2.6	8

na, data not available.

### Identification of CNEs

The public genomes of sequenced diatoms were aligned pairwise against the reference *P. multistriata* genome with Lastz. Utilities from the University of California Santa Cruz (UCSC) genome browser source code tree (Speir *et al*., 2016) were used to generate NET alignments from the raw pairwise alignments.

The pairwise NET alignments in MAF format were combined into a single diatom NET alignment file using the roast binary from the Multiz package (Blanchette *et al*., 2004) with *P. multistriata* as reference. Custom Perl scripts were used to scan the diatom NET alignment to identify conserved intergenic blocks (window, 20 bp; step, 10 bp) which do not overlap gene/expressed sequence tags in the species conserved. Searches for transcription factor binding sites were performed using Jaspar 2014 (Mathelier *et al*., 2014).

### Expansion of gene families in *P. multistriata*

Proteomes of Stramenopiles were compared against hidden Markov models (HMMs) of protein families classified in the SUPERFAMILY database (Wilson *et al*., 2009). The comparison was performed using Perl scripts provided by the SUPERFAMILY database (http://supfam.cs.bris.ac.uk/SUPERFAMILY/howto_use_models.html) and the hmmscan binary from HMMER3 (Eddy, 1995). For each SUPERFAMILY present in *P. multistriata*, a Z‐score was calculated using the following formula (no. of SUPERFAMILY genes in *P. multistriata* – mean no. of SUPERFAMILY genes in all proteomes)/SD of SUPERFAMILY genes in all proteomes.

### Identification of gene families by clustering of protein sequences

An All vs All Blastp search was performed on the combined Fasta file of proteomes from bacteria, archaea and 50 eukaryotes. The results of the Blastp search were provided to the orthAgogue software (Ekseth *et al*., 2014) for the estimation of homology between the protein sequences. The ‘abc’ format output from orthAgogue was given to the MCL software (Enright *et al*., 2002) for clustering of the proteins into homologous groups.

### Estimation of gene family gains and losses in Stramenopiles

Clusters containing only one‐to‐one orthologues of each stramenopile species (85 clusters, considering 13 species mentioned in the ‘Expansion of gene families in *P. multistriata*’ subsection) were chosen to generate the species tree for Stramenopiles using Mafft (Katoh & Standley, 2013).

The alignments were concatenated and trimmed with trimAl. ProtTest (Darriba *et al*., 2011) was then run on the trimmed concatenated alignment to determine the best amino acid substitution matrix to generate a phylogenetic tree based on the Bayesian Information Criterion score. The identified model was employed to generate the phylogenetic tree using a maximum likelihood and a Bayesian approach employing RAxML and MrBayes (Stamatakis, 2006). In both approaches, *Blastocystis hominis* was used as outgroup. Protein clusters with at least one member from any stramenopile species were identified to obtain 28 927 clusters. For each stramenopile species, a binary code was established stating the presence or absence of the species in each cluster. The binary file, together with the maximum likelihood tree, was subjected to Dollo parsimony analysis using the Phylip package (Felsenstein, 1989). The topologies of the maximum likelihood and Bayesian trees were compared with treedist from the Phylip software, indicating an identical topology.

### Identification of genes acquired from red algae and by HGT from bacteria in *P. multistriata*

Identification of HGT events in *P. multistriata* was performed with the following steps: identification of protein clusters containing at least one *P. multistriata* protein; building of a multiple alignment for each cluster using Mafft (Katoh & Standley, 2013); trimming of columns with ≥ 95% gaps in the alignment generated using trimAl (Capella‐Gutiérrez *et al*., 2009); generation of a phylogenetic tree using FastTree.

Phylogenetic trees for each cluster were parsed to identify genes of potential bacterial origin using the following criteria: identification of a clade represented in the majority by bacteria, archaea and diatoms (≥ 90%) without members from metazoa, plantae or fungi; bootstrap cut off at the clade of interest ≥ 0.5 or if the average bootstrap value for the tree is ≥ 0.5 (if one of the bootstrap values is ≤ 0.5, the tree is still retained (if other filters are passed) as a candidate with medium confidence); to add further stringency to the analysis, at least five bacterial members must be present in the clade of interest (10 when *P. multistriata* is the only eukaryote in the clade) to avoid false positives as a result of misplacement of a single protein within the clade of another taxon, which can be caused by issues such as long branch attraction.

### Co‐culture experiments

Three independent co‐culture experiments were performed, two for RNA‐seq (MT+B856xMT‐B939 and MT+B938xMT‐B857) and one for quantitative PCR (MT+B937xMT‐B936). A bipartite glass apparatus (Duran flasks; VWR, Dresden, Germany) (Paul *et al*., 2012) was used for the co‐culture of strains of opposite MTs. A 0.22‐μm pore size hydrophilic polyvinylidene fluoride membrane (Durapore, Millipore) was placed in between the bottles to keep the cells separate. Control parental strains were grown in monoculture. The cell concentration was 80 000 cells ml^−1^ for each strain. The cells were grown in f/2 medium (Guillard, 1975). A 36‐h dark incubation was employed to synchronize the cultures. Samples were collected 2 and 6 h after the start of the experiment. Fifty‐millilitre samples were centrifuged, resuspended in cold methanol and stored at −20°C. They were resuspended in Tris‐EDTA buffer, treated with RNase I (300 μg ml^−1^) for 45 min and stained with SYBR Green (1 : 10 000 dilution of SYBR^®^ Green I – 10 000× concentrate, Invitrogen) for 15 min. Cell cycle synchronization was verified with a FACSCalibur flow cytometer (Becton Dickinson BioSciences Inc., Franklin Lakes, NJ, USA) with standard filters and a 488‐nm Ar laser. SYBR Green fluorescence (DNA) was collected through 530 ± 30‐nm optical filters in order to assess the percentage of cells in the different cell cycle phases. Control cells always presented a bimodal distribution of SYBR Green fluorescence, allowing the assessment of cell cycle blockage (one peak) in treated samples. Sample acquisition was realized using BD CellQuest software, and the relative proportions of cells in the different phases of the cell cycle were assessed using ModFit software (Verity Inc., Palo Alto, CA, USA).

### RNA extraction and sequencing

Samples were collected on 1.2‐μm pore size membrane filters (RAWP04700 Millipore) and extracted with Trizol™ (Invitrogen) according to the manufacturer's instructions; the gDNA contamination was removed by DNase I treatment (Qiagen), followed by purification using an RNeasy Plant Mini Kit (Qiagen). RNA quantity was determined using a Qubit^®^ 2.0 Fluorometer (Life Technologies, Thermofisher, Waltham, MA, USA) and integrity using a Bioanalyzer (2100 Bioanalyzer Instruments, Agilent Technologies, Santa Clara, CA, USA).

Libraries were prepared using a Beckman Biomek FX and an Illumina^®^ TruSeq^®^ Stranded Total RNA Sample Preparation kit, with poly‐A selection and starting with 500 ng of total RNA. Samples were sequenced on an Illumina HiSeq2000 producing single‐end 50‐bp reads. Library preparation and sequencing were performed at the Genecore Facility of the European Molecular Biology Laboratory (EMBL), Germany.

### RNA‐seq filtering, mapping and differential expression analysis

The raw sequencing reads were processed with Trimmomatic (Bolger *et al*., 2014) to trim low‐quality bases and adapters and to filter reads with low quality and smaller than 36 bases. The Star aligner (Dobin *et al*., 2013) was used to map the filtered reads onto the *P. multistriata* genome. The Augustus gene models were associated with the mapped reads from each sample to generate raw counts for each gene as a measure of their expression level. EdgeR (Robinson *et al*., 2010) was used to obtain the differentially expressed genes. In brief, generalized linear models were used to estimate dispersion considering multiple factors (MT, control/sexualized and species strain), whereas a more classical negative binomial distribution was used to compare only the control and sexualized stages independently in each MT.

For quantitative PCR validation, total RNA was extracted from samples collected at 6 h. One microgram of total RNA was reverse transcribed using a QuantiTect^®^ Reverse Transcription Kit (Qiagen). Nineteen genes were selected (Table S1). *TUB‐A* (Adelfi *et al*., 2014) was used as reference. Real‐time PCR amplification and analyses were performed as described in Patil *et al*. (2015).

### Identification of homologous genes and Ka : Ks analysis

The analysed data included 12 152 and 19 703 coding DNA sequences (CDSs) of *P. multistriata* and *Pseudo‐nitzschia multiseries* (Psemu1, downloaded from the Joint Genome Institute (JGI)), respectively. As a first step, a reciprocal best Blast hit (RBH) was used to identify *P. multistriata* and *P. multiseries* orthologues. Only alignments covering at least 30% of *P. multistriata* sequences were retained. The analysis identified 7128 reciprocal best Blast hits. Next, each pair of sequences was aligned with Prank (Löytynoja, 2014) using the empirical codon model, and the alignments were refined using trimAl (Capella‐Gutiérrez *et al*., 2009). Of the 7128 alignments, 6066 were suitable for Ka : Ks calculation. Ka : Ks calculation was performed with KaKs_Calculator (Wang *et al*., 2010); the model for the calculation was chosen for each alignment using the corrected akaike information criterion (AICc) model selection method.

An extended version of the methods can be found in Methods S1.

## Results and discussion

### The *P. multistriata* genome sequence and first identification of CNEs in diatoms

To sequence the genome of *P. multistriata*, we used a strain derived from the cross of two siblings, grown under axenic conditions (Fig. 2a–e). The sequencing and assembly yielded a genome of 59 Mb composed of 1099 scaffolds with an N50 of 139 kb. Estimated heterozygosity was 0.18% and the distribution of allele frequencies peaked at *c*. 0.5, indicating a diploid clonal strain. A total of 99.5% of variable sites presented two alleles; only *c*. 500 of *c*. 110 000 variable sites showed more than two alleles, mainly associated with repeats and noncoding regions. A total of 12 008 genes were predicted on the assembled scaffolds. The regions comprising coding genes accounted for 50% of the genome, where *c*. 80% of genes (9653 genes) were assigned a Uniprot ID and an additional 214 genes were exclusively annotated for the presence of a protein domain. Estimation of genome completion by Cegma identified 221 (89.11%) of CEGs as complete and an additional seven CEGs (3%) as partial, indicating a high‐quality genome assembly and gene build. The statistics and features of the genome assembly and gene prediction for *P. multistriata* and selected Stramenopiles are summarized in Table 1 and Fig. S1.

Sequence conservation at the amino acid level can be a potential indicator of species divergence; hence, we used the sequence homology between species with known evolutionary history to estimate the divergence within diatom genomes (Fig. 2f). Consistent with its known phylogenetic relationships (Kooistra *et al*., 2007), *P. multistriata* shows maximum amino acid identity with the congeneric species *P. multiseries*, followed by the phylogenetically close *F. cylindrus* and then by the more distant *P. tricornutum*. The group of raphid pennates to which the four species belong is thought to have evolved *c*. 60 million yr ago (Ma) (Kooistra *et al*., 2007). Yet, the divergence of these diatom pairs is comparable with that between eukaryotic pairs known to have separated earlier (*Plasmodium falciparum* and *P. vivax*, reported to have separated *c*. 90–100 Ma (Perkins & Schall, 2002); *Arabidopsis thaliana* and *Physcomitrella patens*, with flowering plants reported to have diverged from the bryopsids *c*. 400–450 Ma (Rensing *et al*., 2008)), confirming the rapid evolutionary rates in diatoms (Bowler *et al*., 2008).

In comparison with coding genes, the noncoding part of diatom genomes remains vastly unexplored, with no precise information on noncoding regions that might act as regulatory elements, as reported in animals, plants and unicellular eukaryotes (Vavouri *et al*., 2007; Piganeau *et al*., 2009; Haudry *et al*., 2013). Here, we take a first step towards the identification and classification of CNEs in diatoms using a comparative genomic approach centred on *P. multistriata*. CNEs play a role in the regulation of gene expression, often being part of promoters or enhancers (Woolfe *et al*., 2005). We identified a core set of *c*. 1500 CNEs in the genome of *P. multistriata* (mean length, 110 bp; mean identity, 73%; Table S2) when compared with other diatom genomes. As expected, the majority of the predicted CNEs (93%, 1462 CNEs) were conserved exclusively between the *Pseudo‐nitzschia* species (Fig. 2g). A smaller subset of *c*. 50 CNEs was conserved between *Pseudo‐nitzschia* species and *F. cylindrus* (26), between *P. multistriata* and *P. tricornutum* (15), and between *P. multistriata* and *T. pseudonana* (10, Fig. 2g), suggesting functional constraints leading to noncoding conservation over large evolutionary distances. The predicted CNEs showed a significant enrichment to be located near transcription start sites (TSSs) (Student's *t*‐test, *P* = 0.0034; Fig. S2a), indicating that they are probably involved in transcriptional regulation. Genes associated with the gene ontology (GO) molecular function terms ‘*signal transducer activity*’ and ‘*sequence‐specific DNA binding transcription factor activity*’ were enriched in loci containing CNEs (Fisher test, *P* ≤ 0.05), further supporting the functionality of the CNE. In addition, a significant enrichment of transcription factor binding sites of major transcription factor families was observed in CNE sequences compared with that observed in random sequences of similar size (Fig. S2b–d; Table S3). Thus, the proximity to genes related to the regulation of transcriptional control, together with the binding site propensity for transcription factors, corroborate previous reports which indicate that CNEs are often involved in the regulation of transcription (Inada *et al*., 2003; Sanges *et al*., 2013), and provide evidence in support of their functionality in diatoms.

Approximately 25% of the *P. multistriata* genome comprises repetitive elements, 8% of which are in genic regions and 92% of which are in intergenic regions. The repetitive element coverage is significantly higher than that of other diatoms (6.4% in *P. tricornutum* and 1.9% in *T. pseudonana*). Of the known classes of repetitive elements, LTR retrotransposons were the most abundant group of annotated repeats (6%) (Fig. S3a–c), similar to previous reports in other diatoms (Maumus *et al*., 2009), as well as other Stramenopiles (Cock *et al*., 2010; Ye *et al*., 2015), yeast (Bleykasten‐Grosshans & Neuvéglise, 2011) and some flowering plants (Bennetzen, 2005). To investigate the role of LTRs in shaping the genomic structure of *P. multistriata*, a *de novo* search specific for complete LTR elements was performed in the genomes of five diatoms and that of *Oryza sativa*. Intact retroelements have a built‐in molecular clock useful for estimating their insertion times, based on sister LTR divergence. *Pseudo‐nitzschia* species have, on average, older insertions of LTR elements (45% insertions in the last 0.5 Mya) with respect to *F. cylindrus*,* P. tricornutum* and *T. pseudonana* (80%, 65% and 61% insertions in the last 0.5 Mya, respectively) (Table S4), indicating an earlier expansion of LTR retroelements in the *Pseudo‐nitzschia* genus. However, LTR elements of the Copia lineage also showed a recent burst of activity, suggesting that they might still be active in generating genetic variability in *Pseudo‐nitzschia*, as shown in *P. tricornutum* (Maumus *et al*., 2009) (Fig. S3d).

### High‐resolution phylogenomic analyses define gene family expansions, gene gains and HGT in diatoms

Lineage‐specific gene duplications, losses and pseudogenization, together with genome rearrangements and horizontal transfer of genes between species, have paved the way for the evolution of diversity (Koonin, 2010).

**Figure 3:**
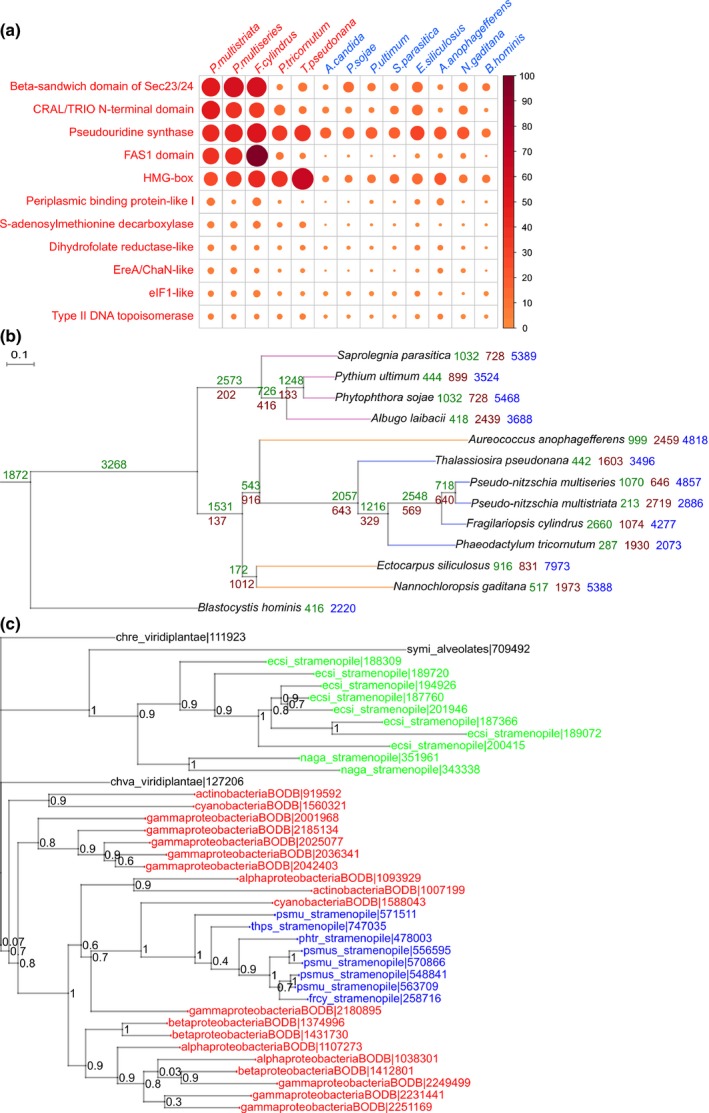
Evolution of gene families in diatoms. (a) Expansion of gene families within diatoms. Each column represents a stramenopile species and each row represents a given gene family showing expansion within diatoms as compared with other Stramenopiles. Names of diatom species are given in red, whereas names of other Stramenopiles are given in blue. The colour intensity and size of the circles are proportional to the number of genes falling under the given gene family. (b) A species tree of Stramenopiles derived using a maximum likelihood approach, built using 85 genes showing one‐to‐one orthology among the selected species. The selected genes include genes with a wide range of functions. Branch lengths are drawn to scale. At each branch point, the number of gene family gains and losses is indicated in green and brown, respectively. The number of orphans present in each organism is shown in blue. (c) Phylogenetic tree for a cluster containing proteins annotated with an uncharacterized cystatin‐like domain, conserved in bacteria. The tree topology depicts a potential horizontal gene transfer event which led to the introduction of the gene within diatoms. The regions coloured red, blue and green represent bacteria, diatoms and other Stramenopiles, respectively. Species codes used in the tree: ecsi, *Ectocarpus siliculosus*; naga, *Nannochloropsis gaditana*; symi, *Symbiodinium minutum*; psmu, *Pseudo‐nitzschia multistriata*; psmus, *Pseudo‐nitzschia multiseries*; frcy, *Fragilariopsis cylindrus*; phtr, *Phaeodactylum tricornutum*; thps, *Thalassiosira pseudonana*; chre, *Chlamydomonas reinhardtii*; chva, *Chlorella variabilis*; BODB suffix is used for all bacterial species. For the correspondence between protein IDs used in this tree and GenBank IDs, see Supporting Information Methods S1.

In order to identify gene family expansions in diatoms, we compared the proteomes of 13 Stramenopiles (including five diatoms) against the collection of gene families from the SUPERFAMILY database. In total, 11 families showed expansion within the diatom lineage (Fig. 3; Table S5); none was *P. multistriata* specific. We confirmed an expansion of a gene family (*pseudouridine synthase*) (Fig. 3a) reported to be expanded in the *P. tricornutum* genome (Bowler *et al*., 2008) and found specific expansion events within the order Bacillariales (Fig. 3a).

In addition to gene family expansions, gene family gains and losses also contribute to the evolution and diversification of species. A study on the *Ectocarpus* genome showed large‐scale gene gains and losses within Stramenopiles, where lineages giving rise to multicellularity were reported to show a high rate of evolution of new gene families (Cock *et al*., 2010). Until a few years ago, the limited availability of sequenced genomes resulted in a lack of resolution to identify gene gain/loss events during the divergence of Stramenopiles. Taking advantage of the latest sequenced genomes, we were able to use *c*. 2 million protein sequences from organisms spanning the tree of life (50 eukaryotes, 1000 bacteria, 170 archaea; Table S6) to infer, at an unprecedented resolution, gene gains/losses in diatoms. Phylogenetic clustering analysis generated *c*. 240 000 clusters of putative homologous proteins, 8113 of which contained 9122 *P. multistriata* proteins. Approximately 26% (2886 singlets + 241 genes in 109 clusters) of the *P. multistriata* proteome was predicted to be orphan (*P. multistriata*‐specific) (Fig. S4), which is slightly less than that reported for other diatoms (*P. tricornutum*, 35%, Bowler *et al*., 2008; *T. pseudonana*, 29%, Armbrust *et al*., 2004) and similar to estimates deriving from transcriptomic data (Di Dato *et al*., 2015). Based on these data, we built a comprehensive species tree for 13 Stramenopiles by concatenating the alignments of 85 clusters of one‐to‐one orthologues conserved across the 13 species. The species tree topologies generated independently by maximum likelihood and Bayesian inference were identical and supported by bootstrap values > 90 at all branch points (Fig. 3b). The tree topology is congruent with a previous report (Yang *et al*., 2012), except for *Aureococcus anophagefferens* and diatoms forming a monophyletic group separated from the other Stramenopiles (*Ectocarpus siliculosus* and *Nannochloropsis gaditana*). Interestingly, a similar topology has been obtained recently using chloroplast genomes to infer phylogeny (Ševčíková *et al*., 2015), suggesting, in accordance with our results, a comparatively more recent split of pelagophytes and diatoms than previously estimated (Gobler *et al*., 2011). We then performed a Dollo parsimony analysis considering the presence/absence profiles of the identified protein clusters within the produced stramenopile species tree. Only those clusters containing at least one stramenopile member were considered (*c*. 27 000 clusters). We observed large‐scale gene family gain and loss events (Fig. 3b), suggesting a high order of genetic diversity among the species compared. A large number of orphan gene families were predicted for each species, confirming extensive species‐specific gains and/or rapid gene divergence as a major feature of stramenopile genomes. We then performed a GO term enrichment analysis on the subset of gene families gained at particular branch points (Figs 3b, S5). Although gene families significantly gained by autotrophic Stramenopiles (lower clade in Fig. 3b) were mostly related to photosynthesis (Fig. S5a), gene families associated with terms such as ‘*mitotic cell cycle spindle assembly check point*’, ‘*synaptonemal complex assembly*’ and ‘*G protein‐coupled receptor signalling pathway*’ were enriched in *Pseudo‐nitzschia* species and *F. cylindrus*, suggesting that potential novel mechanisms evolved in these diatoms to regulate cell division and, possibly, sexual reproduction (Fig. S5b).

The clusters of homologous genes produced were then used for the identification of putative HGT events from bacteria to diatoms. A total of 32% of the *P. multistriata* genes were placed in clusters containing putative orthologues from bacteria, red algae, plants, fungi and metazoans. Among these, genes falling exclusively within clusters dominated by bacterial genes might reflect ancient HGT events from bacteria to diatoms. We identified 392 *P. multistriata* proteins showing homology exclusively with bacteria (Fig. S6). To refine these results, we developed a stringent classification method which, starting from the clusters of orthologous proteins, generated > 9000 phylogenetic trees containing at least one *P. multistriata* protein and, based on the topology of each tree, predicted 252 genes of bacterial origin specifically within diatoms. This is < 50% of the number of genes reported to be of bacterial origin in *P. tricornutum* (587 genes) (Bowler *et al*., 2008) and more than a previous estimate at lower resolution (Lommer *et al*., 2012). Repeating the analysis considering HGT events within Stramenopiles and the SAR supergroup (Stramenopiles, Alveolates and Rhizaria), we predicted 353 and 438 *P. multistriata* genes of potential bacterial origin, respectively. We conclude that the detailed taxonomic resolution introduced in our study allowed for the filtering of candidate genes previously considered to be of bacterial origin via HGT because of a lack of data from related species at the time of analysis. The 252 genes predicted to be of bacterial origin are proposed as a conservative set of genes introduced in the diatom lineage through HGT (Table S7). They are smaller in size than average (*t*‐test; *P* value for gene length, 1e‐04; *P* value for exon length, 1.4e‐05), with no significant difference in the number of exons/gene (Mann–Whitney test, *P* = 0.48) and length of introns (*t*‐test, *P* = 0.24) (Fig. S7). These genes are enriched for GO terms involved in processes such as ‘*energy metabolism*’, ‘*oxidative stress response*’ and ‘*substrate transport*’ (Fig. S8; *P* < 0.05). In support of our results, the ‘*quinone oxidoreductase*’ (PSNMU‐V1.4_AUG‐EV‐PASAV3_0025570.1) was already known to be derived from HGT in diatoms (Nosenko & Bhattacharya, 2007). In addition, a significant difference (*t*‐test, *P* = 0.014) in the GC content for HGT genes compared with all *P. multistriata* genes supports their foreign origin (Garcia‐Vallvé *et al*., 2000) (Fig. S9). Twenty‐four HGT events are specific to the *Pseudo‐nitzschia* genus; an example is shown in Fig. 3(c).

Apart from bacteria, 123 genes in *P. multistriata* were classified to be of red algal origin (Table S8), consistent with the notion that diatom progenitors originated from an ancient secondary endosymbiosis event involving a red alga and a heterotrophic eukaryote (Bowler *et al*., 2010).

### Global gene expression changes at the onset of the sexual phase highlight a stronger response in MT− cells

**Figure 4:**
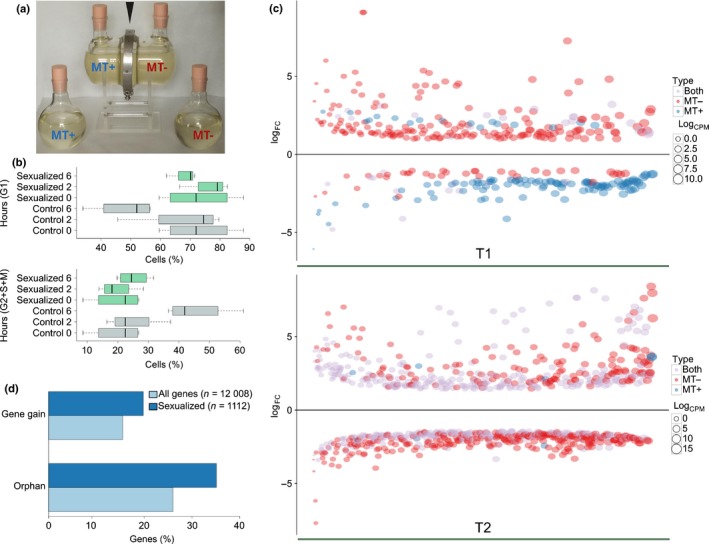
Cell cycle and gene expression changes in the early stages of sexual reproduction. (a) Co‐culture glass apparatus containing cultures of opposite mating type (MT) separated by a membrane held by a metal ring (black arrowhead), and control bottles containing each of the two MTs. (b) Cell cycle phases of MT+ and MT– control (grey) and sexualized (green) samples represented by the relative percentage of cells in G1 (upper) and S + G2 + M (lower) phases, at the beginning of the experiment (0) and 2 and 6 h later. Whiskers in the boxplot extend to ± 1.5 × interquartile range (IQR). (c) Plot showing the log_FC_ (fold change) (*y*‐axis) of genes differentially expressed, ordered according to the log_CPM_ (counts per million) on the *x*‐axis. (d) Percentage of orphan genes and gene gains in the set of genes differentially expressed at the onset of the sexual phase in *Pseudo‐nitzschia multistriata* compared with the percentages of the same classes in the entire gene set.

We exploited the controllable life cycle of *P. multistriata* to investigate the changes occurring in diatom cells in a key phase of their life cycle (Fig. 4). To study gene expression changes induced by the perception of chemical cues deriving from the mating partner, each of two *P. multistriata* strains was placed in one compartment of an apparatus that allowed free exchange of the medium, but not physical contact between the cells (Fig. 4a). Sampling times were 2 h (T1) and 6 h (T2) after co‐culture, the time at which the two MTs are sensing each other (black arrows in Fig. 1; Fig. S10; Table S9). Interestingly, although control strains in an isolated monoculture continued to progress through the cell cycle, cells of both MTs in the experimental set‐up arrested their cell cycle in the G1 phase (Fig. 4b). Previous observations over a longer period of time (14 d, Scalco *et al*., 2014) have revealed a marked decrease in growth of *P. multistriata* cultures undergoing sexual reproduction. The occurrence of growth arrest concomitantly with sexual reproduction is known in yeast, where the cell cycle is arrested in G1 as a consequence of pheromone signalling (Wilkinson & Pringle, 1974). One of the roles of pheromones is indeed cell synchronization to release gametes, simultaneously increasing the success of sexual reproduction (Frenkel *et al*., 2014). A total of 1112 genes (9% of the total *P. multistriata* genes) were differentially expressed in any of the comparisons (all sexualized vs all control samples, MT+ sexualized vs MT+ controls, and MT− sexualized vs MT− controls) (Tables S10, S11). A larger number of genes appeared to be regulated in MT− compared with MT+ (596 in MT− and 182 in MT+, Fisher test, *P* < 0.01) (Table S12). In a cross, both MTs behave similarly (most of the cells arrest their growth and only a small fraction, *c*. 20%, undergo meiosis; Scalco *et al*., 2014), and the gametes produced are morphologically indistinguishable (Fig. 1). However, microscopic and time‐lapse observations have shown that the *P. multistriata* MT− cell in a pair undergoes meiosis, on average, 30 min earlier than the MT+ cell (Scalco *et al*., 2016). This observation suggests that the general response of the cells to pheromones is slightly out of phase, with MT− cells initiating the process earlier than MT+ cells, and this partly explains the different gene expression profiles between MT+ and MT−. In addition to this asynchrony, other MT‐specific changes can be explained by the fact that each of the two MTs secretes specific pheromones, produced by different mechanisms and triggering MT‐specific responses. Signalling involving multiple molecules has been demonstrated for *S. robusta* (Moeys *et al*., 2016) and postulated for *Pseudostaurosira trainorii* (Sato *et al*., 2011). In *S. robusta*, the compound diproline acts as an attraction pheromone (Gillard *et al*., 2013), whereas the molecule SIP+, secreted by MT+ cells, triggers both cell cycle arrest and diproline production in MT− cells (Moeys *et al*., 2016).

### Genes differentially expressed during sexual reproduction are involved in signalling, metabolism, nutrient transport and meiosis

The largest fraction of genes was found to be regulated in both MTs at T2 (Fig. 4c), where we observed a general tendency to downregulate genes encoding nutrient transporters, such as silicate (PSNMU‐V1.4_AUG‐EV‐PASAV3_0083020.1), ammonium, nitrate/nitrite and formate transporters (Tables 2, S10), suggesting that the cells, once the sexual phase is initiated, modulate their nutrient uptake. Interestingly, two *P. multistriata* genes with significant homology to the *P. tricornutum* diatom‐specific cyclins *dsCYC5* and *dsCYC4* were downregulated at T2 (Tables 2, S10). *Phaeodactylum tricornutum dsCYC5* is known to respond to phosphate addition and is supposed to be involved in signal integration to regulate the cell cycle, whereas *dsCYC4* is involved in the perception of growth stimuli (Huysman *et al*., 2013). In our experiment, we used nutrient‐replete medium, and it is unlikely that the cells suffered from nutrient limitation after only 6 h of treatment. Thus, the downregulation of nutrient transporters and cyclins involved in sensing the nutritional status suggests the existence of a complex interplay for the integration of external signals, including mating signals. Upregulation of genes encoding the cohesin complex (*SMC1*,* SMC3*,* SCC3*,* RAD21*; Patil *et al*., 2015), required to hold sister chromatids together during the S phase, indicated preparation for meiosis, which will occur a few hours later (Fig. 1; Scalco *et al*., 2016). This was also supported by the simultaneous upregulation of the meiosis‐related genes *RAD51‐A1*,* RAD51‐C*,* SMC5*,* PSD5* and Smc‐containing proteins (Tables 2, S10).

**Table 2:** Selection of genes differentially expressed in *Pseudo‐nitzschia multistriata* cells 6 h after chemical contact of opposite mating types (MTs)

Pathway/process	GeneModel ID	Gene description	log_FC_
ABC transporters	PSNMU‐V1.4_AUG‐EV‐PASAV3_0056660.1	ATP‐binding cassette, subfamily B (MDR/TAP), member 1	2.14
Cell cycle and meiosis	PSNMU‐V1.4_AUG‐EV‐PASAV3_0056780.1	DNA repair protein RAD51 homologue 1, RAD51‐A1^a^	7.60
	PSNMU‐V1.4_AUG‐EV‐PASAV3_0104040.1	DNA repair protein RAD51 homologue 3, RAD51‐C^a^	2.86
	PSNMU‐V1.4_AUG‐EV‐PASAV3_0089060.1	Pds5^a^	1.95
	PSNMU‐V1.4_AUG‐EV‐PASAV3_0102810.1	Structural maintenance of chromosomes protein 5	2.02
	PSNMU‐V1.4_AUG‐EV‐PASAV3_0079810.1	Structural maintenance of chromosomes protein 3	1.67
	PSNMU‐V1.4_AUG‐EV‐PASAV3_0116990.1	Structural maintenance of chromosomes protein 1	2.60
	PSNMU‐V1.4_AUG‐EV‐PASAV3_0072170.1	Cohesin complex subunit SCC1 (RAD21)	2.07
	PSNMU‐V1.4_AUG‐EV‐PASAV3_0079350.1	Cohesin complex subunit SA‐1/2 (SCC3)	2.49
	PSNMU‐V1.4_AUG‐EV‐PASAV3_0081540.1	G2/mitotic‐specific cyclin S13‐6 (dsCYC5)	−1.66
	PSNMU‐V1.4_AUG‐EV‐PASAV3_0095090.1	Cyclin‐B2‐2 (dsCYC4)	−2.14
Nitrate metabolism	PSNMU‐V1.4_AUG‐EV‐PASAV3_0102470.1	Ferredoxin‐dependent glutamate synthase 2	−1.88
	PSNMU‐V1.4_AUG‐EV‐PASAV3_0012680.1	Ammonium transporter 1, member 5	−2.01
	PSNMU‐V1.4_AUG‐EV‐PASAV3_0048930.1	Nitrate/nitrite transporter NarU	−1.88
Transcription factors	PSNMU‐V1.4_AUG‐EV‐PASAV3_0036500.1	Transcription factor SKN7	−2.63
	PSNMU‐V1.4_AUG‐EV‐PASAV3_0061620.1	Transcriptional activator Myb	−2.31
Protein processing	PSNMU‐V1.4_AUG‐EV‐PASAV3_0032180.1	Major intracellular serine protease	−1.96
Receptor like	PSNMU‐V1.4_AUG‐EV‐PASAV3_0028550.1	Probable leucine‐rich repeat receptor‐like protein kinase At2g33170	2.39
	PSNMU‐V1.4_AUG‐EV‐PASAV3_0087210.1	Probable leucine‐rich repeat receptor‐like protein kinase At1g35710	−1.84
	PSNMU‐V1.4_AUG‐EV‐PASAV3_0079570.1	Receptor‐like protein kinase 5	−1.83
	PSNMU‐V1.4_AUG‐EV‐PASAV3_0113140.1	Probable leucine‐rich repeat receptor‐like serine/threonine protein kinase At4g08850	−1.70
	PSNMU‐V1.4_AUG‐EV‐PASAV3_0039440.1	Receptor‐like protein kinase HSL1	−1.69
	PSNMU‐V1.4_AUG‐EV‐PASAV3_0029550.1	Leucine‐rich repeat receptor‐like serine/threonine protein kinase GSO2	−1.62
	PSNMU‐V1.4_AUG‐EV‐PASAV3_0018920.1	Somatic embryogenesis receptor kinase 1	−1.52
	PSNMU‐V1.4_AUG‐EV‐PASAV3_0055410.1	Probable leucine‐rich repeat receptor‐like serine/threonine protein kinase At4g26540	−2.32
	PSNMU‐V1.4_AUG‐EV‐PASAV3_0055070.1^b^	Probable leucine‐rich repeat receptor‐like protein kinase	−2.41
	PSNMU‐V1.4_AUG‐EV‐PASAV3_0055080.1^b^		
Signalling	PSNMU‐V1.4_AUG‐EV‐PASAV3_0051110.1	Soluble guanylate cyclase 88E	6.30
Miscellaneous	PSNMU‐V1.4_AUG‐EV‐PASAV3_0102760.1	Protein aardvark (adhesion protein)	6.09
	PSNMU‐V1.4_AUG‐EV‐PASAV3_0112340.1	Tetratricopeptide (TPR) repeat‐containing protein^c^ (protein–protein interactions)	2.94
	PSNMU‐V1.4_AUG‐EV‐PASAV3_0063230.1	Salicylate carboxymethyltransferase	3.50
	PSNMU‐V1.4_AUG‐EV‐PASAV3_0078620.1	E3 ubiquitin‐protein ligase Nedd‐4	3.30
MT‐specific			
Miscellaneous	PSNMU‐V1.4_AUG‐EV‐PASAV3_0041130.1	Heat shock factor protein 3	3.41
	PSNMU‐V1.4_AUG‐EV‐PASAV3_0103000.1	Cathepsin D	6.96
	PSNMU‐V1.4_AUG‐EV‐PASAV3_0067710.1	TPR repeat‐containing protein^c^ (protein–protein interactions)	−2.86

a Gene identity defined in Patil *et al*. (2015).

b Gene models to be merged in a single model.

c Automatic annotation yields nephrocystin.

One of the strongest inductions (6.3‐fold) was observed in both MTs for a soluble guanylate cyclase (Tables 2, S10). Notably, a bifunctional guanylyl cyclase/phosphodiesterase (*GC/PDE*) was found to be upregulated in MT− *S. robusta* cells in response to the SIP+ pheromone (Moeys *et al*., 2016). Although the *S. robusta GC/PDE* and the *P. multistriata* guanylate cyclase show some degree of similarity (25% identity), they are not orthologues (data not shown). The *P. multistriata GC/PDE* homologue (PSNMU‐V1.4_AUG‐EV‐PASAV3_0076150.1) was also regulated, albeit at a lower level (1.9‐fold). Therefore, through different genes, cyclic guanosine monophosphate (cGMP) synthesis and downstream activation of signalling are common responses to pheromone perception.

The presence of G protein‐coupled receptors (GPCRs), one of the largest families of cell surface receptors in eukaryotes, has been reported in *P. multiseries* (Port *et al*., 2013), and we also detected gain of this class in *P. multistriata*. In the RNA‐seq data, PSNMU‐V1.4_AUG‐EV‐PASAV3_0072880.1, homologous to *GPCR3* (Port *et al*., 2013), is upregulated, and the inositol phospholipid signalling pathway, which is downstream of GPCRs, also appears to be employed by *P. multistriata* cells to transduce information (Table S10). GPCRs bind many signalling molecules and our data indicate that they might be involved in the perception of mating cues in diatoms, as has been reported in yeast (Alvaro & Thorner, 2016).

The MT− sexualized strain displayed a seven‐fold increase in Cathepsin D, a pepsin‐like aspartate protease, which has been shown to cleave proteins in the extracellular matrix (Handley *et al*., 2001). In *Saccharomyces cerevisiae*, an extracellular peptidase is involved in the degradation of the pheromone, a process that helps to align the pheromone gradient to detect the direction of the nearest mating partner, increasing mating efficiency (Barkai *et al*., 1998).

Nineteen genes were selected for quantitative PCR validations on samples from an independent experiment, and changes were confirmed for 16 of them (Table S1).

### A subset of genes differentially expressed during sexual reproduction shows lineage‐specific evolution

Rapid divergence and positive selection are common for genes involved in sexual reproduction and can contribute to the establishment of reproductive isolation (Swanson & Vacquier, 2002). Our results support this assumption. A significantly higher proportion of genes differentially expressed during the early phases of sexual reproduction (sensing, Fig. 1) were predicted to be *P. multistriata* orphans (35%) or gene gain events in Bacillariales (20%) with respect to the same proportions in the whole proteome (Fisher test, *P* < 0.05; Fig. 4d; Tables S13, S14). In addition, when comparing the average identity between *P. multistriata* and *P. multiseries* orthologues (or between *P. multistriata* and other diatoms), genes differentially expressed in our experiment showed lower conservation with respect to the entire *P. multistriata* gene set (Fig. S11).

**Figure 5:**
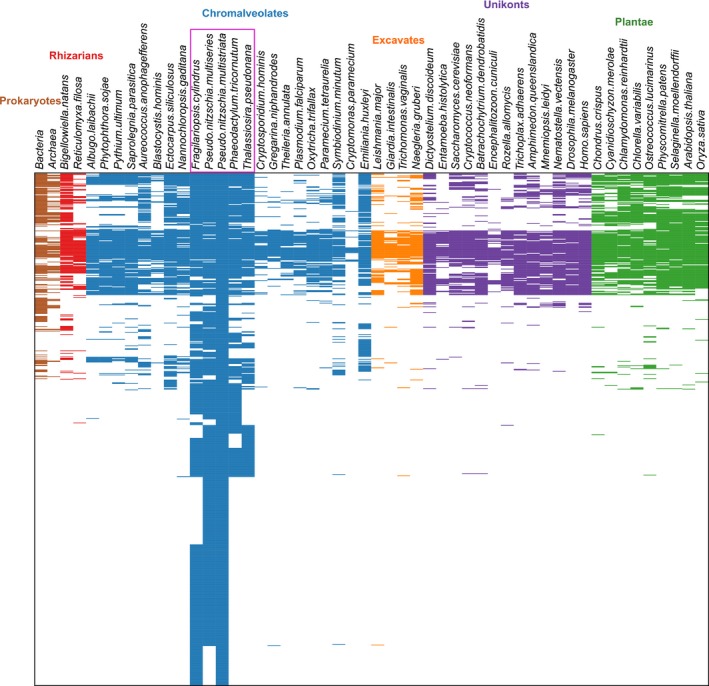
Conservation of the genes differentially expressed in the experiments described in this work. Conservation is shown as the presence/absence of a horizontal line in 52 different species belonging to Prokaryotes, Rhizarians, Chromalveolates, Excavates, Unikonts and Plantae.

Furthermore, using our in‐depth clustering of *P. multistriata* genes with a diverse range of prokaryotes and eukaryotes, we observed that the majority of non‐orphan genes differentially expressed during the sexual phase were specific to diatoms (Figs 5, S11e).

The data indicate that a substantial fraction of the differentially expressed genes are diatom specific, Bacillariales specific or *Pseudo‐nitzschia* specific, or orphans, consistent with the uniqueness of the diatom life cycle and with the necessity to evolve species‐specific mechanisms to attract and mate with the right partner.

Reproductive proteins show a tendency to be under positive selection (Clark *et al*., 2006). In order to identify the *P. multistriata* genes which are under positive selection, we selected all one‐to‐one homologues between *P. multistriata* and *P. multiseries*, and calculated the Ka : Ks ratio (number of nonsynonymous mutations/number of synonymous mutations) to measure their evolutionary divergence (Yang & Bielawski, 2000). Ka : Ks > 1 indicates a selective advantage to amino acid substitutions in a protein. Of the 6066 homologous pairs identified (Table S15), 434 were among those regulated during sexual reproduction and included 11 genes showing a strong positive selection (Ka : Ks > 1). This group contains six unknown genes, two peptidases, two leucine‐rich repeat (LRR) receptor‐like protein kinases and a putative DNA helicase (Table S15). The next gene in the list, with a Ka : Ks value of 0.92, is the homologue of the *S. robusta GC/PDE*. Further studies will clarify whether any of these genes has a specific role in recognizing the right mating partner, avoiding interspecies breeding. Finally, 20 differentially expressed genes are derived from bacteria by HGT in diatoms (Table S16); an example (nitrate/nitrite transporter, PSNMU‐V1.4_AUG‐EV‐PASAV3_0048930.1) is shown in Fig. S12.

**Figure 6:**
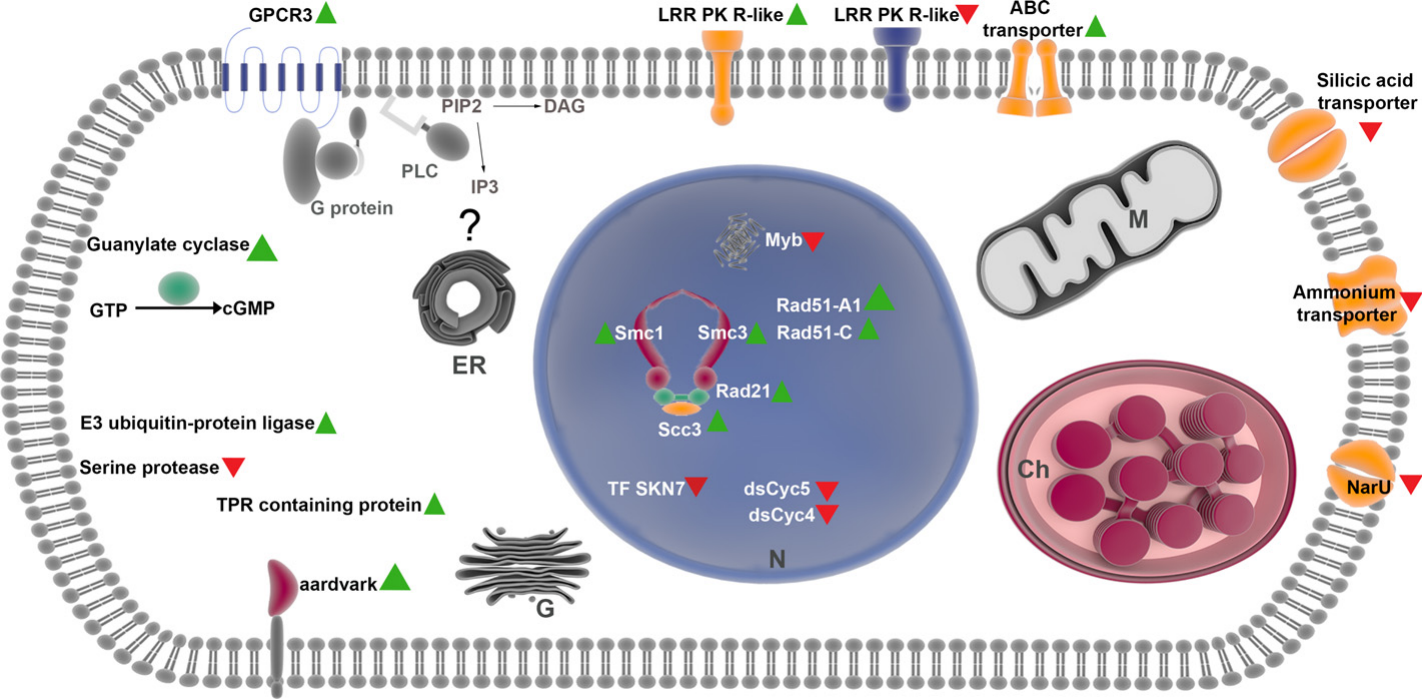
Cell response to sexual cues. Diagrammatic representation of a *Pseudo‐nitzschia multistriata* cell with the principal genes involved in the response to chemical cues acting at the beginning of sexual reproduction. Green triangles represent upregulation and red triangles downregulation of expression. PLC, Phospholipase C; DAG, diacylglycerol; PIP2, phosphatidylinositol biphosphate; IP3, inositol trisphosphate; GTP, Guanosine‐5′‐triphosphate; N, nucleus; ER, endoplasmic reticulum; M, mitochondrion; Ch, chloroplast; G, Golgi; LRR, leucine‐rich repeat.

A schematic summary of the regulated pathways and functions in a *P. multistriata* cell responding to sexual cues is shown in Fig. 6.

These data provide markers for data mining of metatranscriptomic datasets and will improve our ability to understand and monitor toxic *Pseudo‐nitzschia* blooms.

## Author contributions

M.I.F. coordinated the project. M.I.F., R.S., M.M. and M.C. designed the study. S.P., C.F. and R.C. performed the experiments. S.B., S.P., D.M., M.T.R., L.V., F.M., T.M., R.C., R.S. and M.I.F. analysed the data. S.B., M.I.F., R.S. and M.M. wrote the paper with contributions from all authors. All authors read and approved the final manuscript.

## Supporting information

Please note: Wiley Blackwell are not responsible for the content or functionality of any Supporting Information supplied by the authors. Any queries (other than missing material) should be directed to the *New Phytologist* Central Office.

**Fig. S1** General statistics of the *Pseudo‐nitzschia multistriata* genome assembly.
**Fig. S2** Putative association of conserved noncoding elements in *Pseudo‐nitzschia multistriata* with regulation of transcription.
**Fig. S3** Coverage of repeat elements and estimation of long terminal repeat (LTR) insertion period in the *Pseudo‐nitzschia multistriata* genome.
**Fig. S4** Comparison of the number of protein clusters (potential gene families) in chromalveolates, unikonts, plants and prokaryotes (archaea + bacteria) in relation to the clusters with at least one representative from *Pseudo‐nitzschia multistriata*.
**Fig. S5** Enrichment of gene ontology (GO) molecular function terms for gene families gained in photosynthetic Stramenopiles or in *Pseudo‐nitzschia* and *Fragilariopsis*.
**Fig. S6** Comparison of the number of *Pseudo‐nitzschia multistriata* proteins sharing common clusters (potential gene families) with red algae, plants, fungi, metazoans and bacteria.
**Fig. S7** General statistics of *Pseudo‐nitzschia multistriata* genes against those predicted to be of bacterial origin specifically in diatoms.
**Fig. S8** Enrichment of gene ontology (GO) molecular function terms for genes of potential bacterial origin specific to diatoms, Stramenopiles or SAR (Stramenopiles, Alveolates and Rhizaria).
**Fig. S9** GC content of genes acquired by horizontal gene transfer from bacteria as compared with all genes in *Pseudo‐nitzschia multistriata*.
**Fig. S10** Experimental set‐up for gene expression studies at the onset of sexual reproduction.
**Fig. S11** Conservation of genes predicted to be differentially expressed during sexual reproduction in *Pseudo‐nitzschia multistriata* compared with the same data for the entire *P. multistriata* gene set.
**Fig. S12** Screenshot of the *Pseudo‐nitzschia multistriata* genome browser.
**Methods S1** Supplementary methods.

**Table S1** Validation of a selected subset of genes differentially expressed during sexual reproduction in *Pseudo‐nitzschia multistriata* by quantitative PCR
**Table S4** Insertion period estimation of complete long terminal repeats (LTRs) identified in diatom genomes
**Table S8** Annotation for diatom genes of red algal origin
**Table S9** Summary statistics of RNA‐seq read mapping results for *Pseudo‐nitzschia multistriata* samples
**Table S12** Statistics of genes differentially regulated during the sexualized stage in both mating types at two different time points

**Table S2** Genomic coordinates of the *Pseudo‐nitzschia multistriata* conserved noncoding elements, together with coordinates in other diatom species, where each element remains conserved

**Table S3** The core, plant and fungal transcription factor families which show enrichment of binding sites on the *Pseudo‐nitzschia multistriata* conserved noncoding elements

**Table S5** Number of proteins from stramenopile genomes represented by different superfamilies from the SUPERFAMILY database

**Table S6** Details of eukaryotic and prokaryotic organisms considered for the generation of the protein clusters for the *Pseudo‐nitzschia multistriata* proteome

**Table S7** Annotation for the *Pseudo‐nitzschia*/diatom/stramenopile/SAR (Stramenopile, Alveolates and Rhizaria)‐specific genes of bacterial origin

**Table S10** Differential expression analyses of all sexualized samples vs all control samples, MT+ sexualized samples against MT+ controls, and MT− sexualized samples against MT− controls, at two different time points

**Table S11** Log_FC_ (fold change) and false discovery rate (FDR) values for all *Pseudo‐nitzschia multistriata* transcripts for the same conditions as in Table S10

**Table S13** Differentially expressed genes predicted to be gene gain events in diatoms post‐divergence from *Phaeodactylum tricornutum*

**Table S14** Differentially expressed genes predicted to be orphan genes in *Pseudo‐nitzschia multistriata*

**Table S15** Rate of evolution of homologous pairs of *Pseudo‐nitzschia multistriata* and *Pseudo‐nitzschia multiseries*

**Table S16** Genes predicted to be introduced via horizontal gene transfer (HGT) in diatoms, showing differential expression during sexual reproduction in *Pseudo‐nitzschia multistriata*
