# Supplementary material for: Finding a partner in the ocean: molecular and evolutionary bases of the response to sexual cues in a planktonic diatom
Source: New Phytol. 2017 Apr 21;215(1):140–56. doi: 10.1111/nph.14557 (PMC5485032; doi:10.1111/nph.14557)
Supplement: Supplementary file 1 — Fig. S1 General statistics of the Pseudo‐nitzschia multistriata genome assembly. Fig. S2 Putative association of conserved noncoding elements in Pseudo‐nitzschia multistriata with regulation of transcription. Fig. S3 Coverage of repeat elements and estimation of long terminal repeat (LTR) insertion period in the Pseudo‐nitzschia multistriata genome. Fig. S4 Comparison of the number of protein clusters (potential gene families) in chromalveolates, unikonts, plants and prokaryotes (archaea + bacteria) in relation to the clusters with at least one representative from Pseudo‐nitzschia multistriata. Fig. S5 Enrichment of gene ontology (GO) molecular function terms for gene families gained in photosynthetic Stramenopiles or in Pseudo‐nitzschia and Fragilariopsis. Fig. S6 Comparison of the number of Pseudo‐nitzschia multistriata proteins sharing common clusters (potential gene families) with red algae, plants, fungi, metazoans and bacteria. Fig. S7 General statistics of Pseudo‐nitzschia multistriata genes against those predicted to be of bacterial origin specifically in diatoms. Fig. S8 Enrichment of gene ontology (GO) molecular function terms for genes of potential bacterial origin specific to diatoms, Stramenopiles or SAR (Stramenopiles, Alveolates and Rhizaria). Fig. S9 GC content of genes acquired by horizontal gene transfer from bacteria as compared with all genes in Pseudo‐nitzschia multistriata. Fig. S10 Experimental set‐up for gene expression studies at the onset of sexual reproduction. Fig. S11 Conservation of genes predicted to be differentially expressed during sexual reproduction in Pseudo‐nitzschia multistriata compared with the same data for the entire P. multistriata gene set. Fig. S12 Screenshot of the Pseudo‐nitzschia multistriata genome browser. Methods S1 Supplementary methods. [file NPH-215-140-s001.pdf]

New Phytologist **Supporting Information**

Article title: Finding a partner in the ocean: molecular and evolutionary bases of the response to sexual cues in a planktonic diatom

Authors: Swaraj Basu[1], Shrikant Patil[1], Daniel Mapleson[2], Monia Teresa Russo[1], Laura Vitale[1], Cristina Fevola[1], Florian Maumus[3], Raffaella Casotti[1], Thomas Mock[4], Mario Caccamo[2], Marina Montresor[1], Remo Sanges[5], Maria Immacolata Ferrante[1]

The following Supporting Information is available for this article:

Fig. S1 General statistics of the *P. multistriata* genome assembly. **a)** Genome coverage of CDS, intron, UTRs and intergenic regions. **b)** Most represented proteins domains (top 20) in the predicted gene set. **c)** Most represented GO Biological process terms (top 20) in the predicted gene set. **d)** Most represented GO Molecular function terms (top 20) in the predicted gene set. **e)** Most represented GO Cellular component terms (top 20) in the predicted gene set. **f**, **g**) Blobtools outputs, showing the distributions of read coverage, GC, and sequence similarity using data from overlapping paired end library ope2. In **f**, a TAGC plot of the complete *P. multistriata* whole genome sequencing dataset. The x-axis shows the GC content of individual scaffolds plotted against their read coverage (y-axis; logarithmic scale). The scaffold color represents the taxonomic order of their best homology match in the NCBI nt database (with E-value cutoff < 1e-05), those without an annotation are in gray. Bars on the left in **g** indicate the percentage of unmapped and mapped sequencing data of library ope2 to the genome assembly. Bars on the right show the best homology matches in the NCBI nt database (with E-value cutoff < 1e-05).

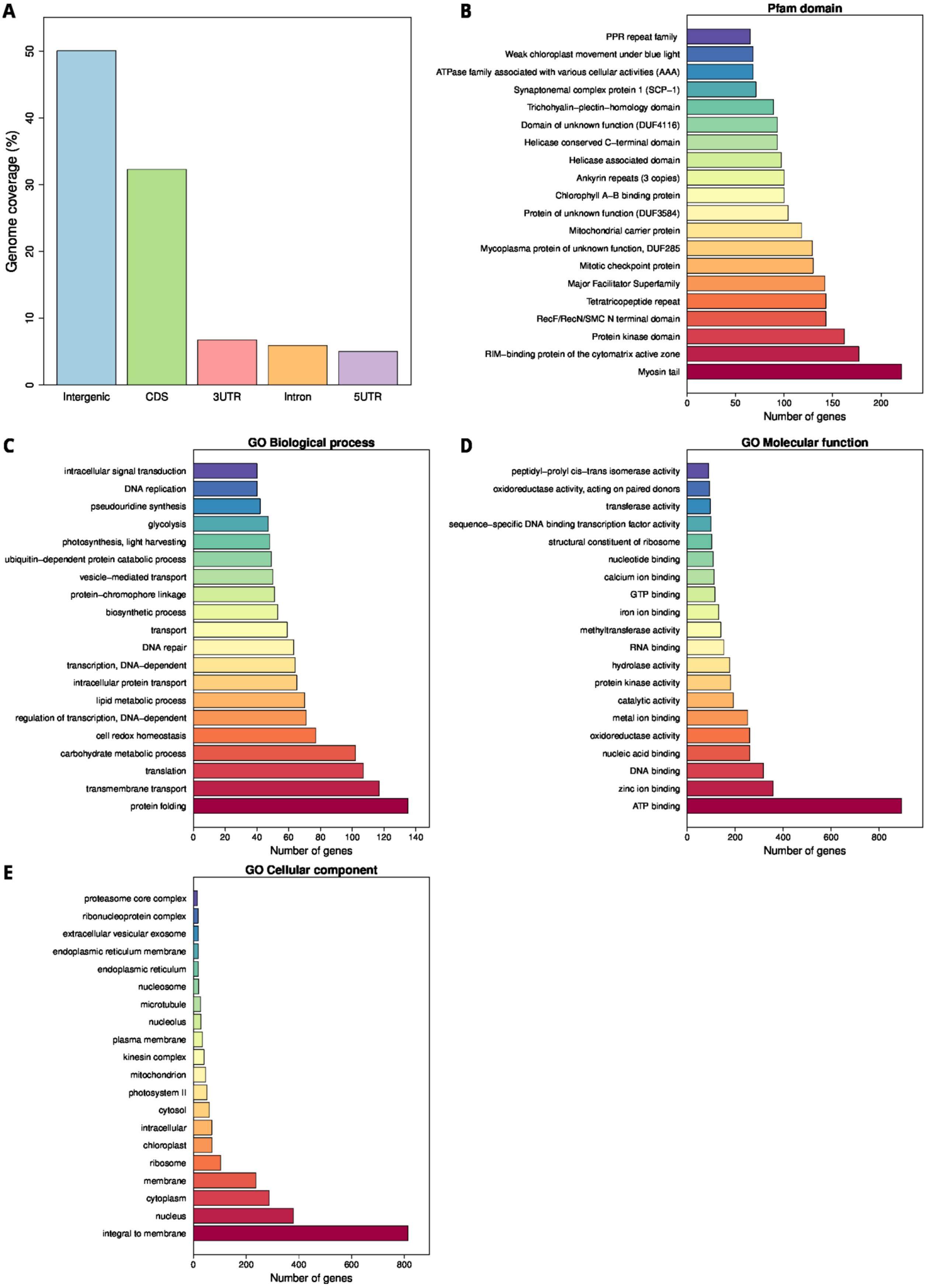

A
Genome coverage (%)
0
10
20
30
40
50
Intergenic
CDS
3UTR
Intron
5UTR
B
Pfam domain
PPR repeat family
Weak chloroplast movement under blue light
ATPase family associated with various cellular activities (AAA)
Synaptonemal complex protein 1 (SCP−1)
Trichohyalin−plectin−homology domain
Domain of unknown function (DUF4116)
Helicase conserved C−terminal domain
Helicase associated domain
Ankyrin repeats (3 copies)
Chlorophyll A−B binding protein
Protein of unknown function (DUF3584)
Mitochondrial carrier protein
Mycoplasma protein of unknown function, DUF285
Mitotic checkpoint protein
Major Facilitator Superfamily
Tetratricopeptide repeat
RecF/RecN/SMC N terminal domain
Protein kinase domain
RIM−binding protein of the cytomatrix active zone
Myosin tail
0
50
100
150
200
Number of genes
C
GO Biological process
intracellular signal transduction
DNA replication
pseudouridine synthesis
glycolysis
photosynthesis, light harvesting
ubiquitin−dependent protein catabolic process
vesicle−mediated transport
protein−chromophore linkage
biosynthetic process
transport
DNA repair
transcription, DNA−dependent
intracellular protein transport
lipid metabolic process
regulation of transcription, DNA−dependent
cell redox homeostasis
carbohydrate metabolic process
translation
transmembrane transport
protein folding
0
20
40
60
80
100
120
140
Number of genes
D
GO Molecular function
peptidyl−prolyl cis−trans isomerase activity
oxidoreductase activity, acting on paired donors
transferase activity
sequence−specific DNA binding transcription factor activity
structural constituent of ribosome
nucleotide binding
calcium ion binding
GTP binding
iron ion binding
methyltransferase activity
RNA binding
hydrolase activity
protein kinase activity
catalytic activity
metal ion binding
oxidoreductase activity
nucleic acid binding
DNA binding
zinc ion binding
ATP binding
0
200
400
600
800
Number of genes
E
GO Cellular component
proteasome core complex
ribonucleoprotein complex
extracellular vesicular exosome
endoplasmic reticulum membrane
endoplasmic reticulum
nucleosome
microtubule
nucleolus
plasma membrane
kinesin complex
mitochondrion
photosystem II
cytosol
intracellular
chloroplast
ribosome
membrane
cytoplasm
nucleus
integral to membrane
0
200
400
600
800
Number of genes

F

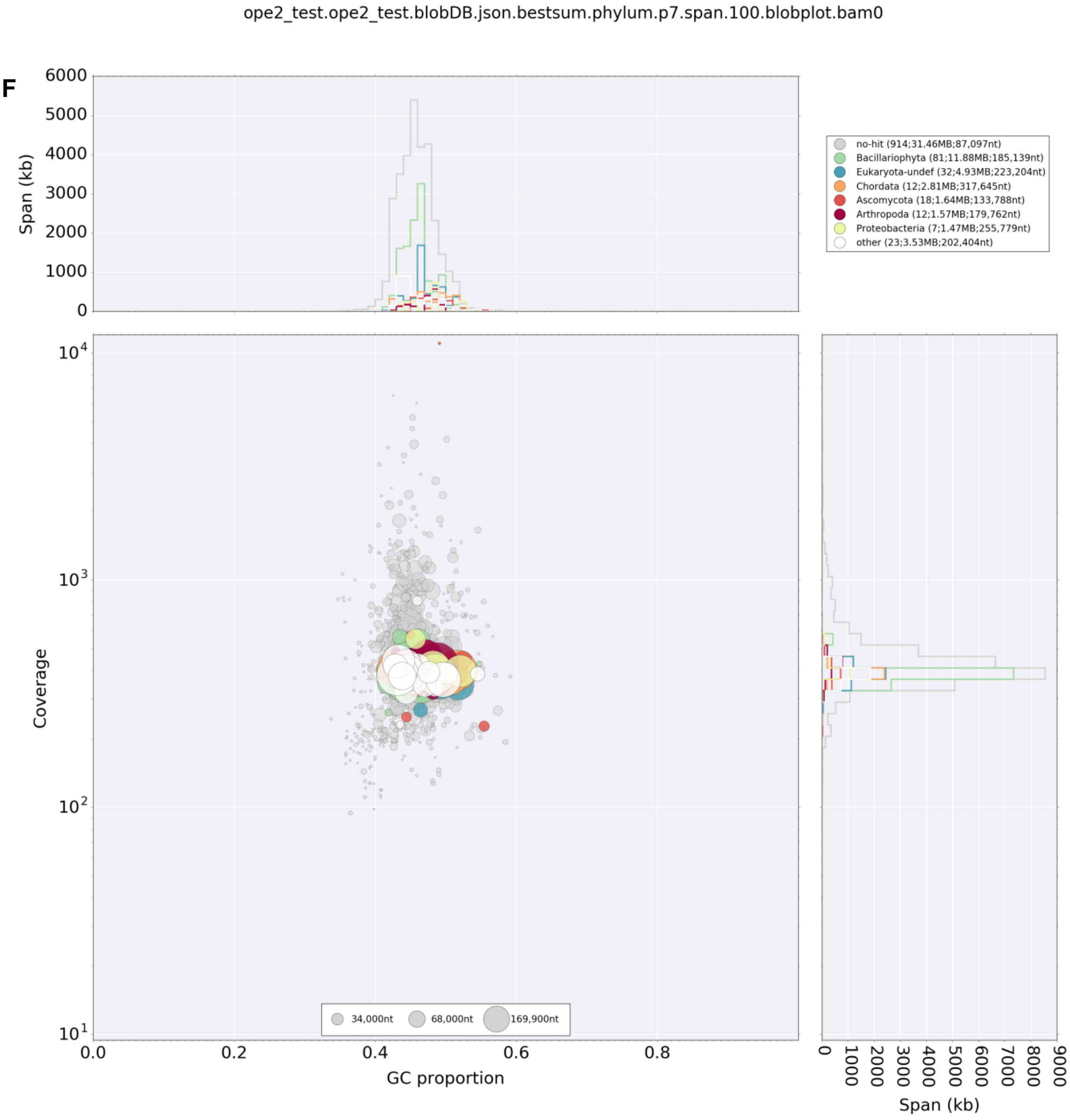

ope2_test.ope2_test.blobDB.json.bestsum.phylum.p7.span.100.blobplot.bam0
Span (kb)
no-hit (914;31.46MB;87,097nt)
Bacillariophyta (81;11.88MB;185,139nt)
Eukaryota-undef (32;4.93MB;223,204nt)
Chordata (12;2.81MB;317,645nt)
Ascomycota (18;1.64MB;133,788nt)
Arthropoda (12;1.57MB;179,762nt)
Proteobacteria (7;1.47MB;255,779nt)
other (23;3.53MB;202,404nt)
Coverage
34,000nt
68,000nt
169,900nt
GC proportion
Span (kb)

G

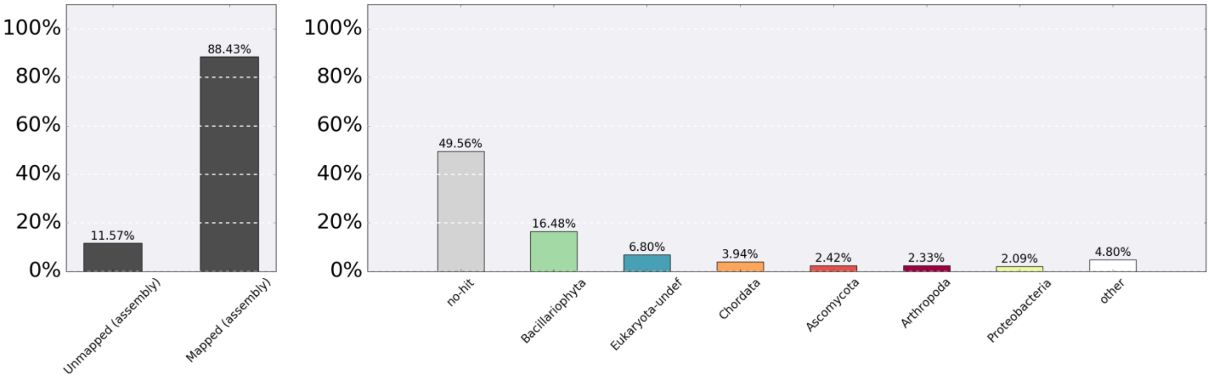

11.57%
88.43%
Unmapped (assembly)
Mapped (assembly)
49.56%
16.48%
6.80%
3.94%
2.42%
2.33%
2.09%
4.80%
no-hit
Bacillariophyta
Eukaryota-undef
Chordata
Ascomycota
Arthropoda
Proteobacteria
other

Fig. S2 Putative association of conserved non-coding elements (CNEs) in *P. multistriata* with regulation of transcription. **a)** Relative distance of conserved non-coding elements from proximal transcriptional start site (TSS) and random genomic regions. The cumulative distribution of the number of transcription factors with a binding site in a set of given sequence features (CNEs and random intergenic regions) using **b)** Core set of transcription factors **c)** Fungal-specific transcription factors, from the JASPAR database and **d)** Plant-specific transcription factors.

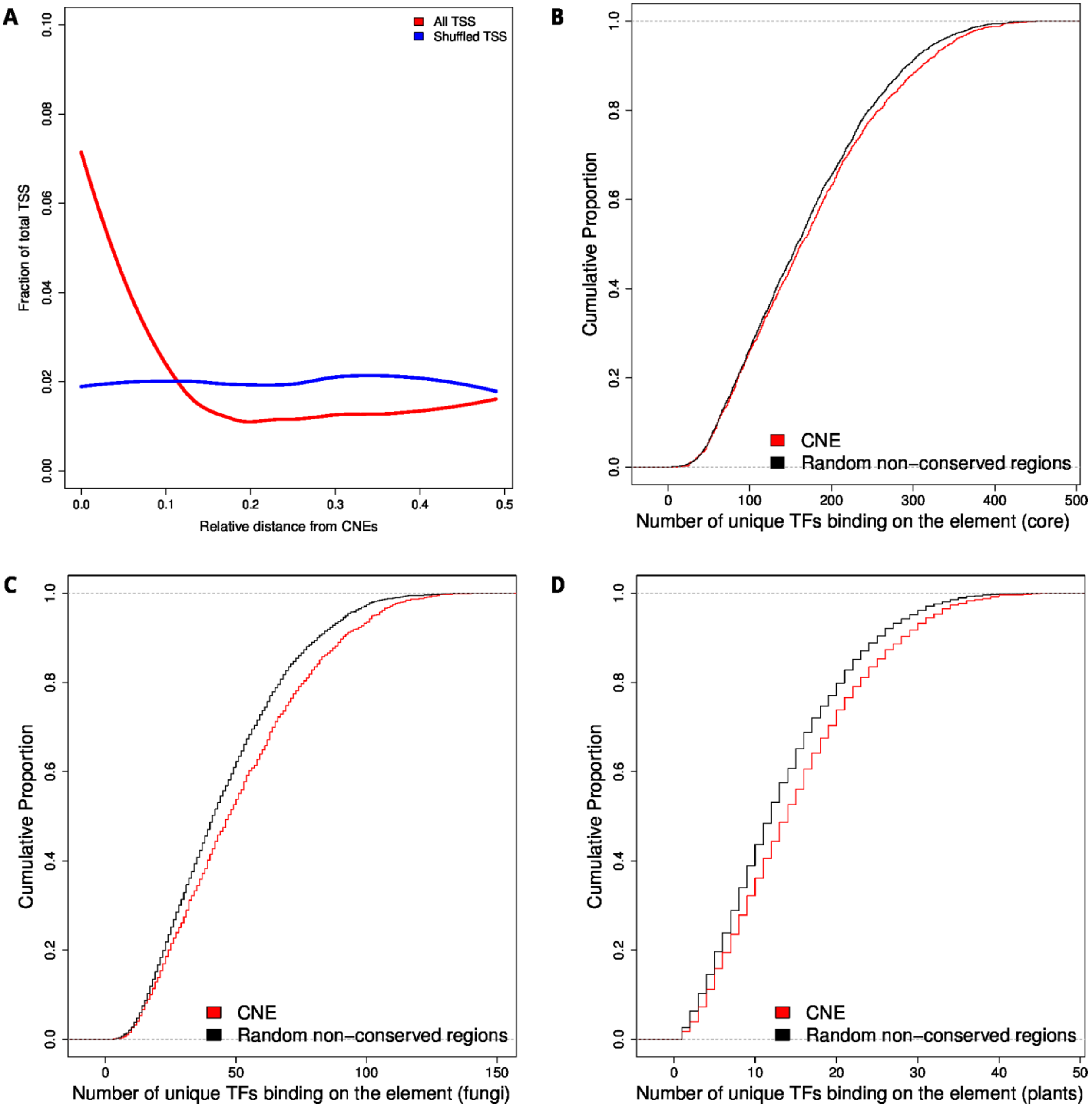

Fig. S3 Coverage of repeat elements and estimation of LTR insertion period in the *P. multistriata* genome. **a)** All repeat classes **b)** LTR elements **c)** DNA transposons **d)** Distribution of LTR insertion time (in million years) for Copia and Gypsy elements in *P. multistriata*.

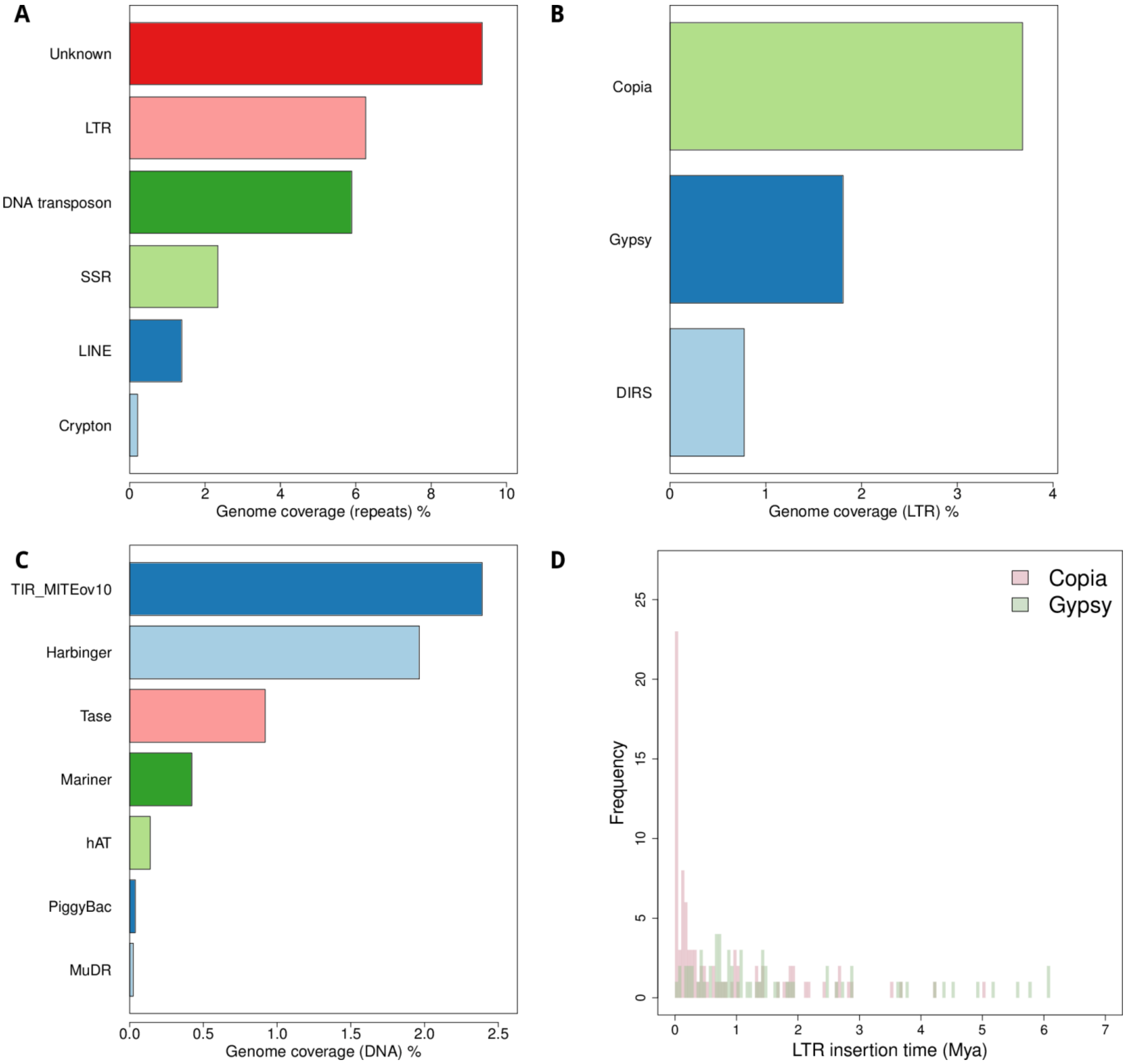

Fig. S4 Comparison of the number of protein clusters (potential gene families) in Chromalveolates, Unikonts, Plantae and Prokaryotes (archaea + bacteria) in relation to the clusters with at least one representative from *P. multistriata.*

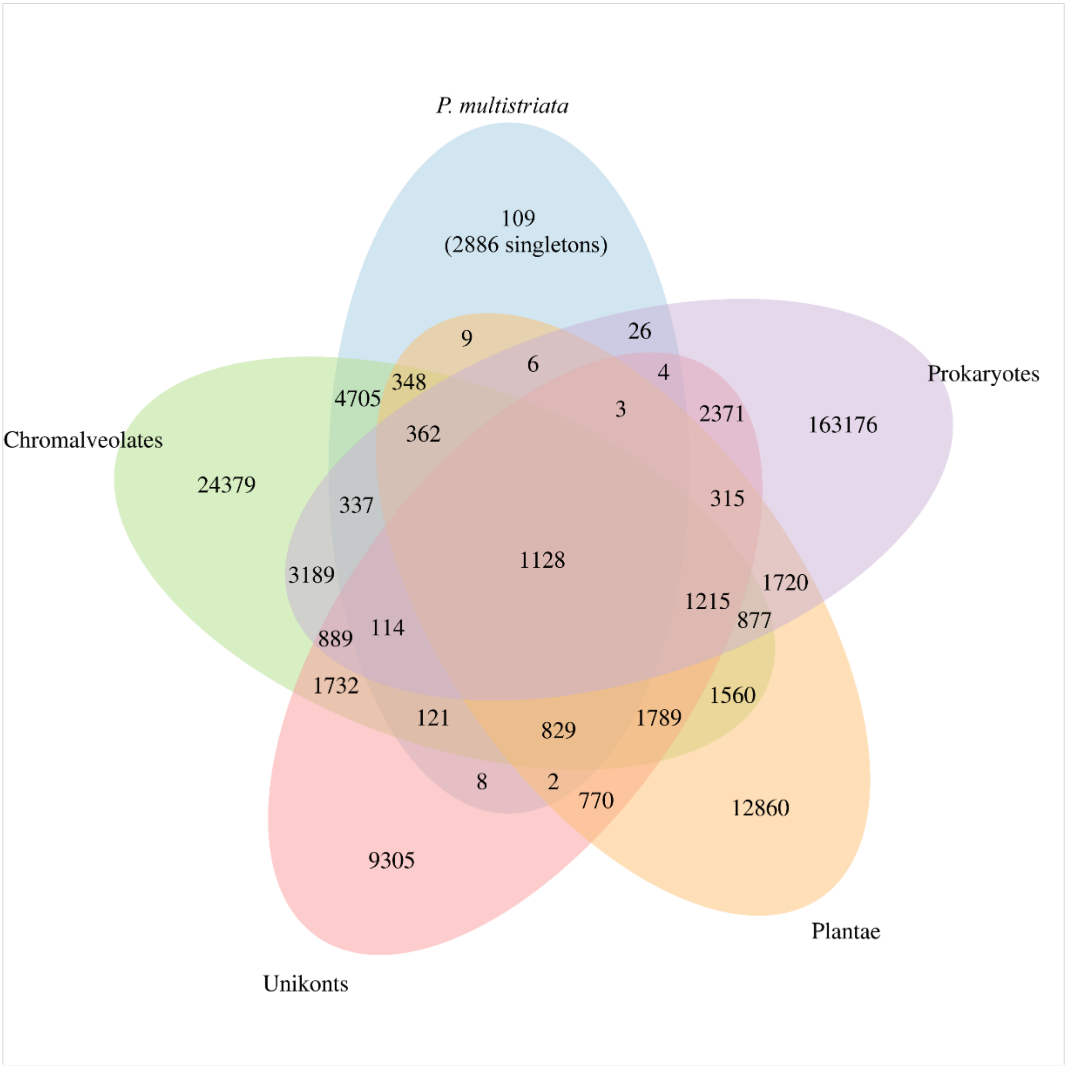

Fig. S5 Enrichment of GO molecular function terms for gene families gained in **a)** photosynthetic Stramenopiles **b)** *Pseudo-nitzschia* and *Fragilariopsis*. The x-axis represents the percentage of genes represented by a given GO term. The y-axis represents the GO terms.

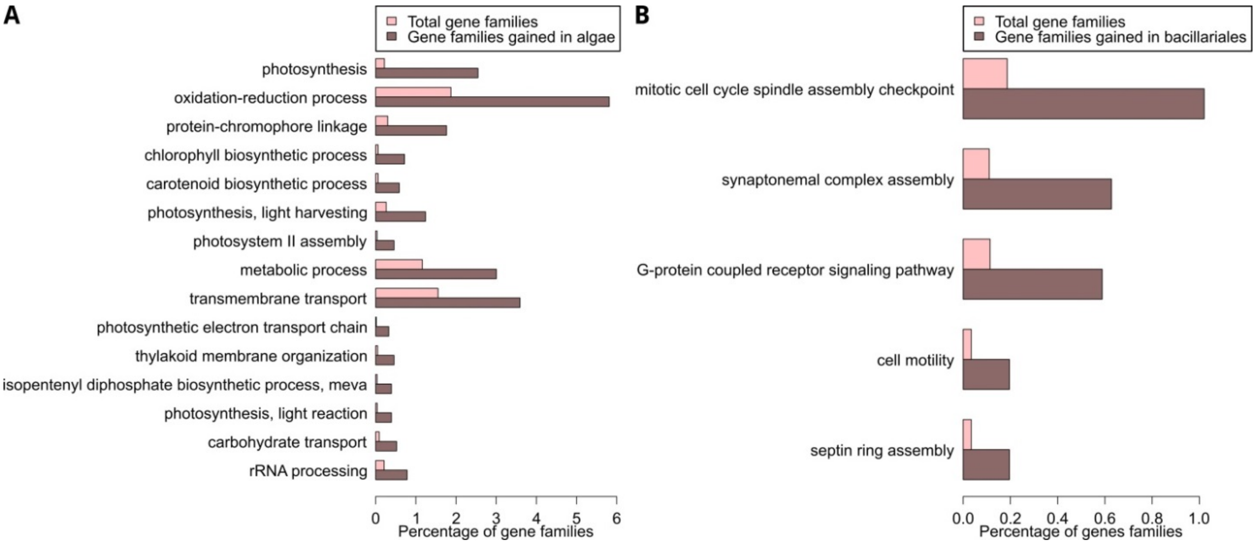

Fig. S6 Comparison of the number of *P. multistriata* proteins sharing common clusters (potential gene families) with red algae, plants, fungi, metazoans and bacteria.

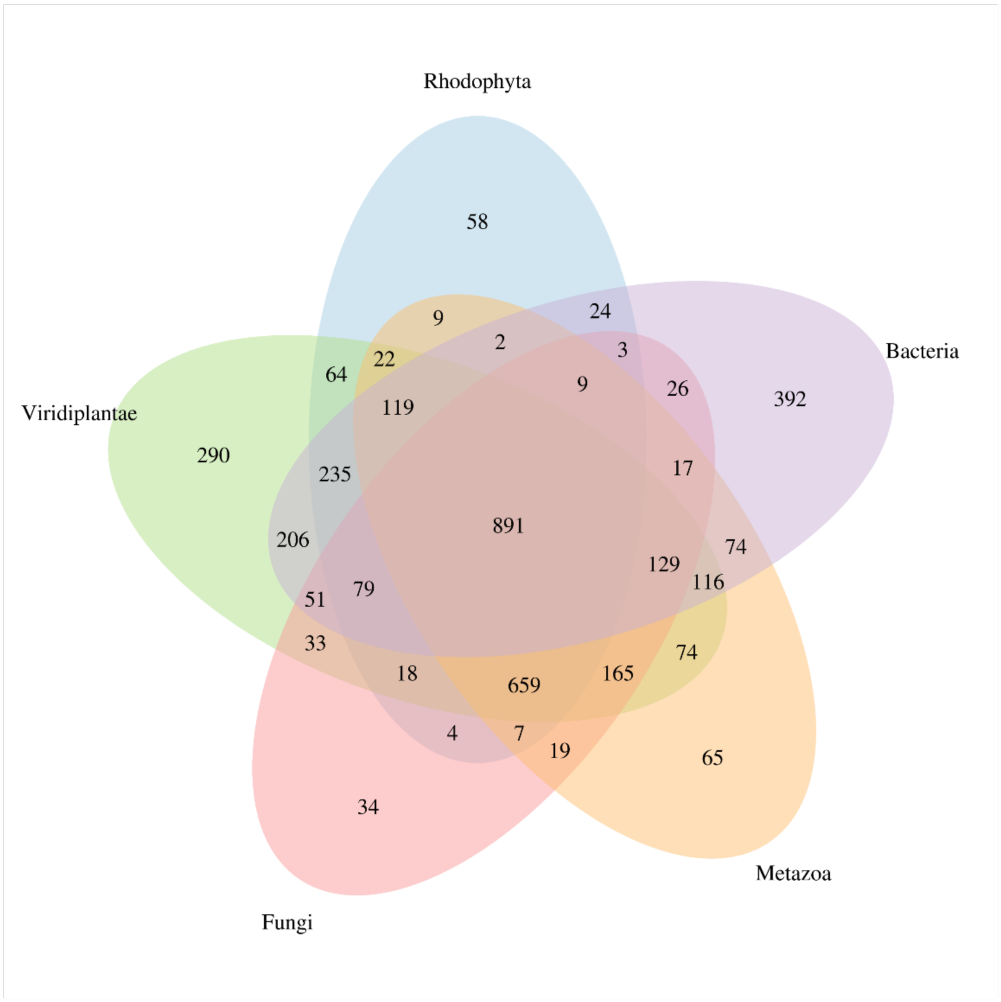

Fig. S7 General statistics of *P. multistriata* genes against those predicted to be of bacterial origin specifically in diatoms. **a)** Gene length **b)** Number of exons per gene **c)** Average exon length per gene **d)** Average intron length per gene. Whiskers in boxplot extend to ±1.5 × IQR (Inter Quartile Range).

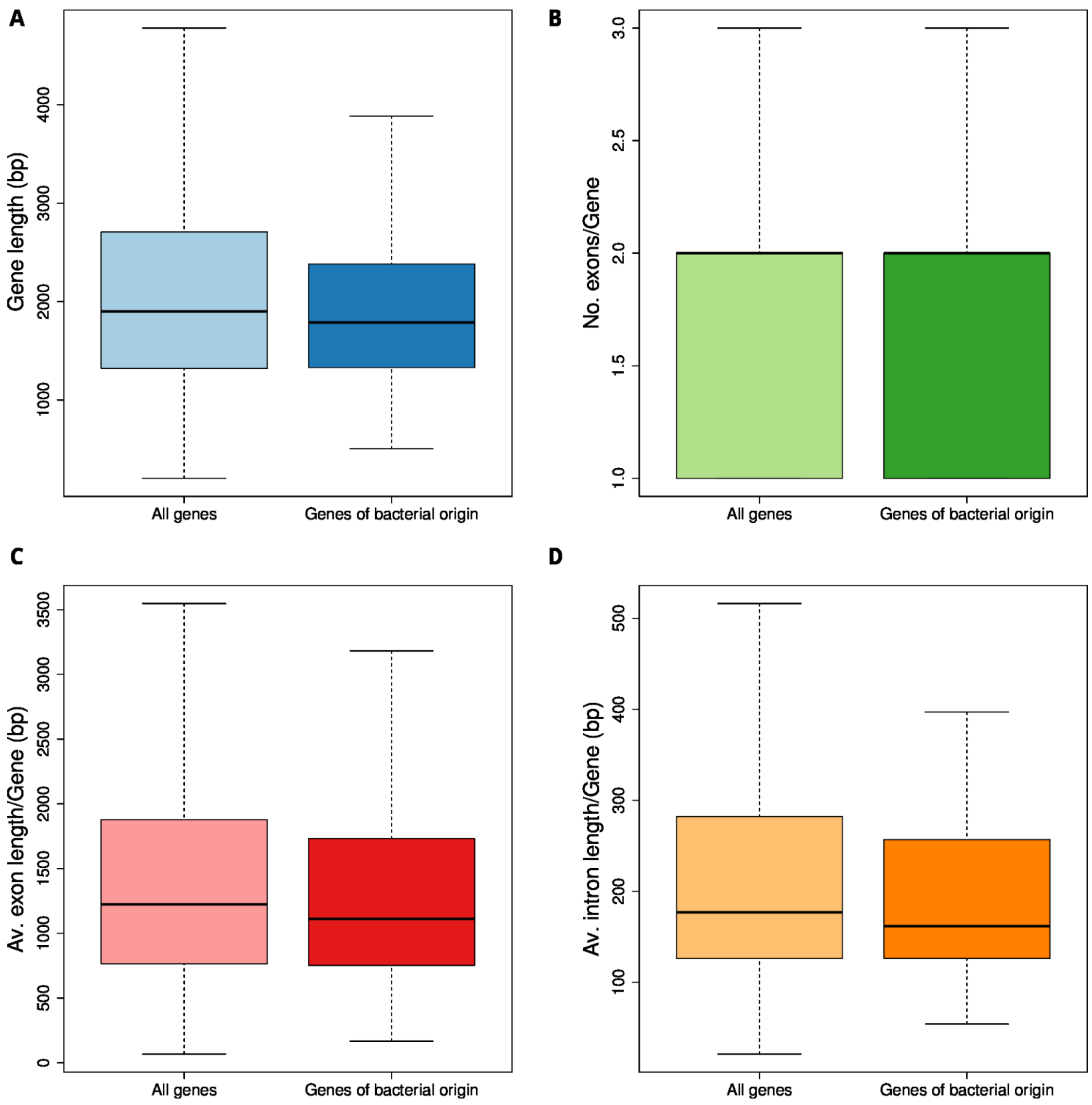

Fig. S8 Enrichment of GO molecular function terms for genes of potential bacterial origin specific to **a)** diatoms **b)** Stramenopiles **c)** SAR supergroup. The x-axis represents the percentage of genes represented by a given GO term. The y-axis represents the GO terms.

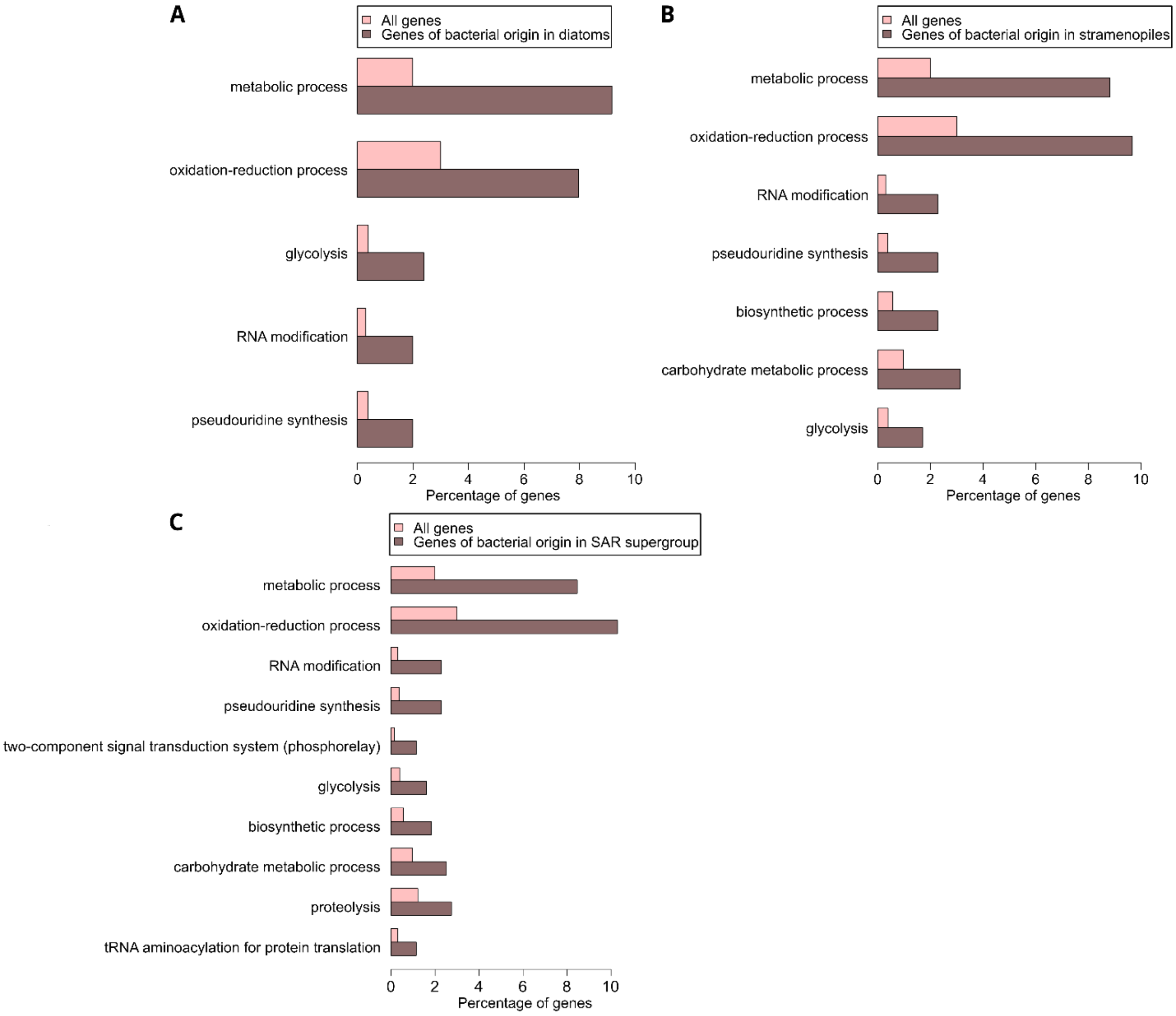

Fig. S9 GC content of genes acquired by horizontal gene transfer from bacteria as compared to all genes in *P. multistriata*

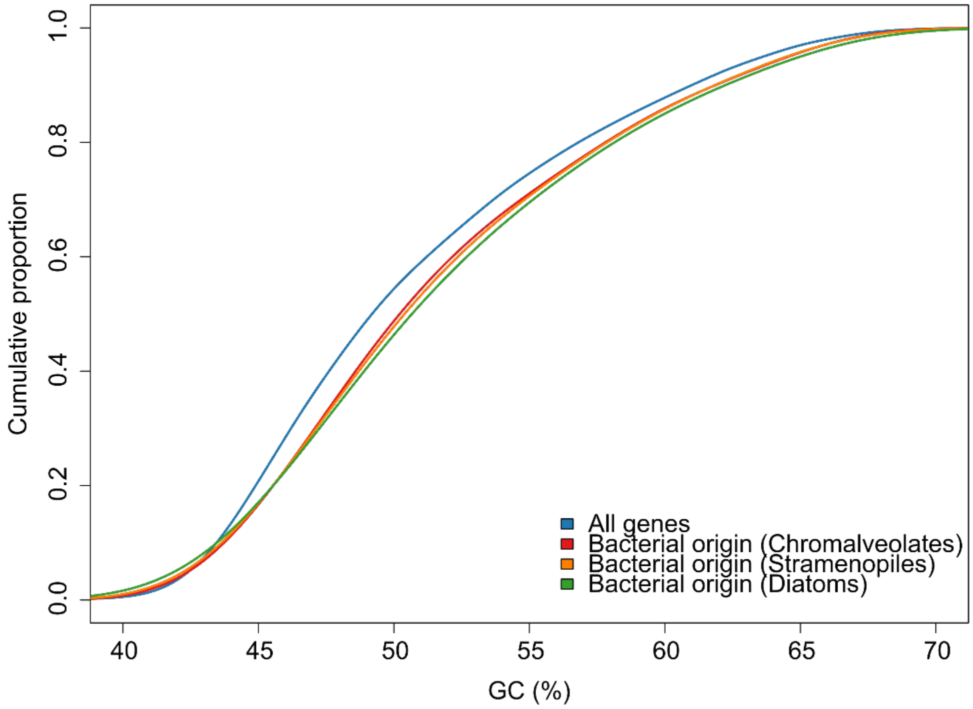

Fig. S10 Experimental set-up for gene expression studies at the onset of sexual reproduction.

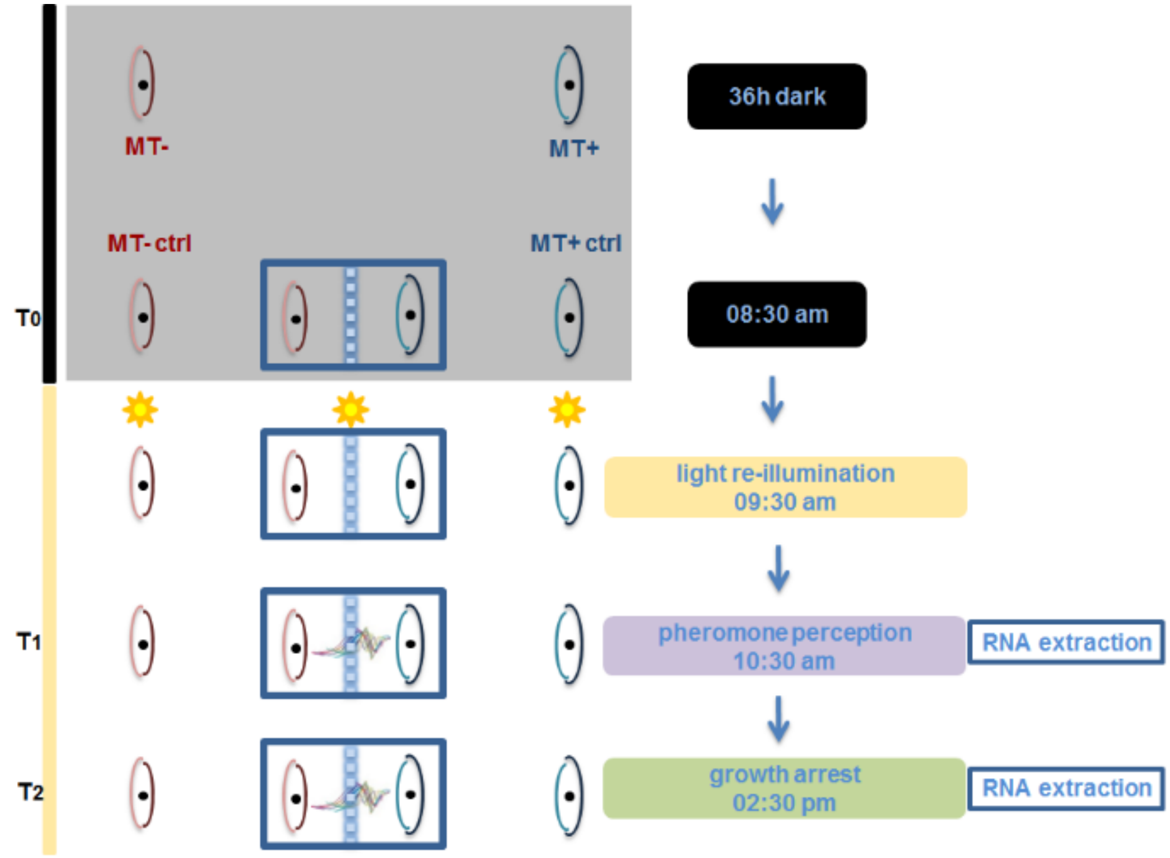

Fig. S11 Conservation of genes (exonic regions) predicted to be differentially expressed during sexual reproduction in *P. multistriata* (green) compared to the same data for the entire *P. multistriata* gene set (light blue). **a)** MULTIZ conservation score between *Pseudo-nitzschia* species. **b)** MULTIZ conservation score between *P. multistriata* and *F. cylindrus.* **c)** MULTIZ conservation score between *P. multistriata* and *P. tricornutum.* **d)** MULTIZ conservation score between *P. multistriata* and *T. pseudonana.* **e)** Percentage of differentially expressed genes with homologs in principal eukaryotic taxa and bacteria. Whiskers in boxplot extend to ±1.5 × IQR (Inter Quartile Range).

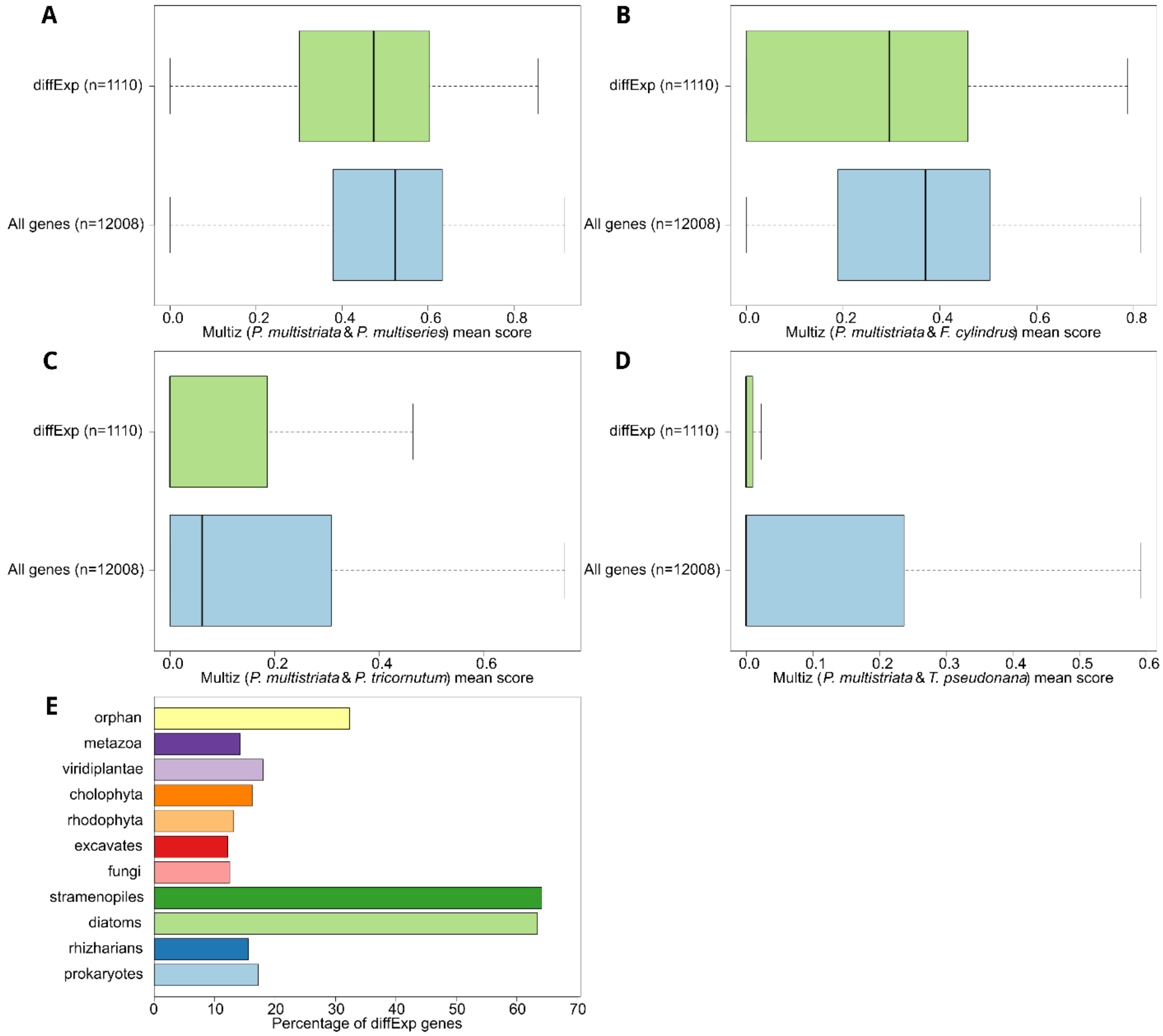

Fig. S12 Screenshot of the *P. multistriata* genome browser. The screenshot shows part of the scaffold 234 containing the gene model PSNMU-V1.4_AUG-EV-PASAV3_0048930.1 (blue bar), with a track showing conservation between *P. multistriata* and *P. multiseries* and tracks showing RNA-seq reads from MT+ control and MT- sexualised.

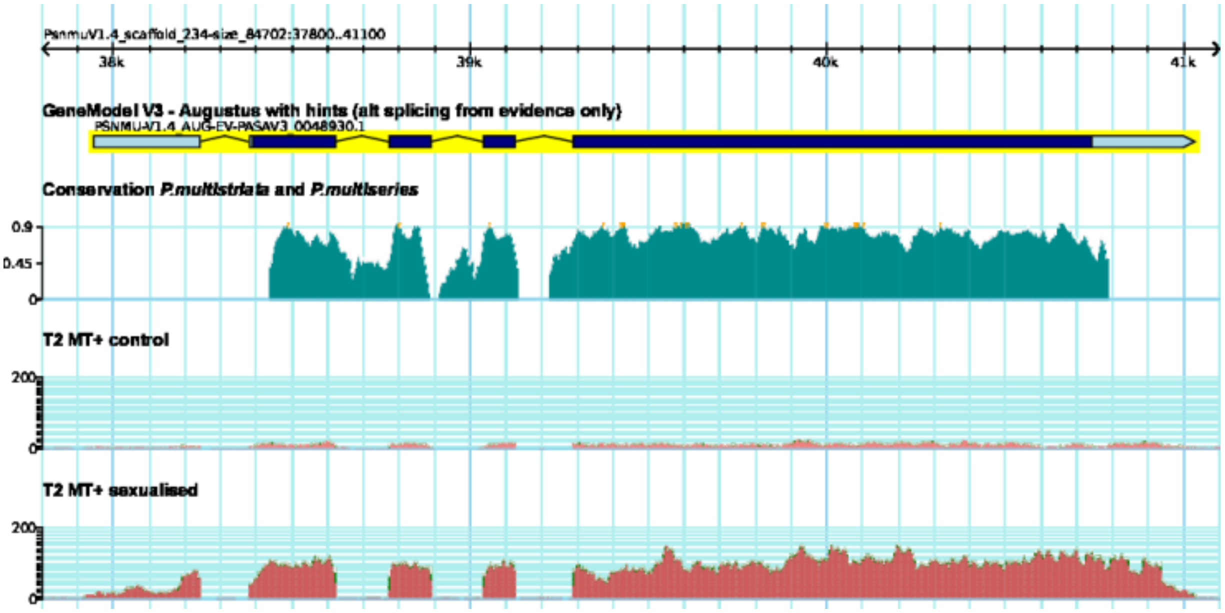

Table S1. Validation of a selected subset of genes differentially expressed during sexual reproduction in *P. multistriata* by qPCR.

Table S2. Genomic coordinates of the *P. multistriata* conserved non-coding elements along with coordinates in other diatom species where each element remains conserved.

Table S3. The core, plant and fungal transcription factor families which show enrichment of binding sites on the *P. multistriata* conserved non-coding elements.

Table S4. Insertion period estimation of complete LTRs identified in diatom genomes.

Table S5. Number of proteins from stramenopile genomes represented by different superfamilies from the SUPERFAMILY database.

Table S6. Details of eukaryotic and prokaryotic organisms considered for generating the protein clusters for *P. multistriata* proteome.

Table S7. Annotation for *Pseudo-nitzschia*/ diatom/ stramenopile/ SAR -specific genes of bacterial origin.

Table S8. Annotation for diatom genes of red algal origin.

Table S9. Summary statistics of RNA-seq reads mapping results for *Pseudo-nitzschia multistriata* samples.

Table S10. Differential expression analyses of i) all sexualized samples versus all control samples, ii) MT+ sexualized samples against MT+ controls and iii) MT- sexualized samples against MT- controls, at two different time points.

Table S11. LogFC and FDR values for all *P. multistriata* transcripts for the same conditions as in Table S10.

Table S12. Statistics of genes differentially regulated during sexualized stage in both mating types at two different time points.

Table S13. Differentially expressed genes predicted to be gene gain events in diatoms post divergence from *P. tricornutum*.

Table S14. Differentially expressed genes predicted to be orphan genes in *P. multistriata*.

Table S15. Rate of evolution of homologous pairs of *P. multistriata* and *P. multiseries.*

Table S16. Genes predicted to be introduced via HGT in diatoms, showing differential expression during sexual reproduction in *P. multistriata*.

Methods S1

Antibiotic treatment to produce axenic cultures

1 ml of exponentially growing culture was inoculated in a medium containing final concentrations of 0.1 mg ml^{-1} Streptomycin (Sigma Aldrich, Saint Louis, MO, US), 0.1 mg ml^{-1} Penicillin (Sigma Aldrich, Saint Louis, MO, US) and 0.5 mg ml^{-1} Ampicillin (Roche, Basel, Switzerland) and allowed to grow for 5-6 days under standard growth conditions. Bacterial contamination was checked in two ways: i) by staining DNA with DAPI and examining cultures under the microscope to check for the presence/absence of bacterial nucleoids; ii) by performing peptone tests. For DAPI staining, 1 µl of DAPI stock solution (4',6-diamidino-2-phenylindole, 1 mg ml^{-1}, Roche, Basel, Switzerland) was added to 1 ml of formalin preserved culture, incubated for 10 minutes and observed under the epifluorescence microscope. For peptone tests, 1 ml of diatom culture was added to a tube containing a peptone solution (1 mg ml^{-1}), incubated in the dark and checked after 2-3 days and 1-2 weeks, growth of bacteria in the tubes indicated contamination. If bacterial contamination persisted, the treatment was repeated. Large volume cultures used for DNA extraction were grown with antibiotics and the contamination tests were always performed on an aliquot of the culture.

DNA extraction

Axenic *Pseudo-nitzschia* cells (strain B856) were collected onto 1.2 µm RAWP membrane filter (Millipore, Billerica, MA, US). The filter was rinsed with 1.5 ml seawater and cells were further collected into eppendorf tubes and pelleted by centrifugation at 3,800 ***g*** at 4 ^{0}C for 5 minutes. The DNA was extracted following a Phenol-Chloroform extraction method (Sabatino *et al.*, 2015) with slight modifications that include cell disruption by adding 400 mg of 0.2-0.3 mm diameter silica beads and vortex mixing at 30 hertz for 85 seconds (3 times), cooling the pellet on ice between the vortex mixing. The extracted DNA was ethanol precipitated, air dried, dissolved in 50 µl of sterile water and stored at -20 ^{0}C until sequencing.

Gene prediction and annotation

Protein-coding genes were predicted by using a workflow that incorporated RNA-seq reads, homologous proteins from *Phaeodactylum tricornutum, Thalassiosira pseudonana* and a *de novo Pseudo-nitzschia multistriata* transcriptome assembly produced (see below). RNA-seq reads were combined from four different libraries, two for sample B856 (libraries HCUO, SRX1070748 and HCUH, SRX1070747), and two for sample B857 (libraries HCUN, SRX1070749 and HATT, SRX1070750), and then passed through a pipeline to use as training data for Augustus (Stanke *et al.*, 2006). To create the training set, the RNA-seq reads were normalised and assembled into transcripts via Trinity (Grabherr *et al.*, 2011) in genome guided mode. Because the *Pseudo-nitzschia* genome appears to be relatively gene dense we used the "jaccard clip" option in Trinity to reduce the number of chimera/fusion transcripts produced. To the training set we also added the *de novo* generated transcriptome assembled with Trinity without the support of the genome using six libraries available on the JGI website (http://genomeportal.jgi.doe.gov/pages/dynamicOrganismDownload.jsf?organism=PsenittraphaseII, and R. Sanges unpublished). A gene model was created by passing the *de novo* and genome guided transcripts into PASA (Haas *et al.*, 2003), and then coding regions were identified using TransDecoder (included in Trinity package), producing 12,933 genes and 20,880 transcripts. The gene model was filtered to 1,182 high-quality, full-length, non-similar (i.e. unique) transcripts. Of these, 982 were used to train an Augustus model and 200 were used for testing. The model built and validated on these high confidence transcripts was then applied to the entire repeat masked assembly, along with external support from homologous proteins aligned using Exonerate (Slater & Birney, 2005), taking into account also repeats and RNA-seq expression levels to predict and annotate all the transcripts from the whole genome. The predicted gene model sequences were further annotated with the Annocript pipeline (Musacchia *et al.*, 2015). The pipeline employs multiple programs to annotate query sequences. The BLASTx program (parameters: word_size = 4, maximum e-value = 10^{-5}, num_descriptions = 5, num_alignments = 5, threshold = 18) is used to annotate the transcriptome against the Swiss-prot (SP) and UniRef90 databases. Further a rpsBLAST search (Camacho *et al.*, 2009)(parameters: maximum e-value = 10^{-5}, num_descriptions = 20, num_alignments = 20)

against profile matrices of the Conserved Domains Database (CDD) is used to predict the protein domains in the transcriptome. Mapping of GO functional classification and the Enzyme Commission IDs and descriptions is performed by using the SwissProt or UniRef accession of the best match for each transcript and the mapping tables from the UniProt distribution and the Expasy database downloaded from the following addresses:
-UniProt:
ftp://ftp.uniprot.org/pub/databases/uniprot/current_release/knowledgebase/idmapping/idmappingselected.tab.gz
-Enzyme: ftp://ftp.expasy.org/databases/enzyme/enzyme.dat
Finally, the dna2pep and Portrait software are used to predict the longest ORF and the non-coding potential score (NCP) of each sequence in the transcriptome. The transcription start sites (TSS) of ab-initio predicted gene models were obtained using the flankBed (-l 1 -r 0 -s) binary from the BEDTools package v2.22 (Quinlan & Hall, 2010).

Assessment of heterozygosity

The raw sequencing reads were aligned to the reference genome using the BWA aligner v (Li & Durbin, 2009). The aligned reads in BAM format were parsed using samtools v1.3.1 (Li *et al.*, 2009) (mpileup --skip-indels -d 250 -m 1 -E) and bcftools v1.3.1 (Li *et al.*, 2009)(call --skip-variants indels --multiallelic-caller --variants-only -O v) to call variants in VCF format. The raw variant calls were further processed using bcftools to filter low quality variants (filter -e 'GT="0/0" || %QUAL<20 || %MAX(DV)<=10') and obtain general statistics on heterozygosity (stats -s).

Repeat Annotation

Repeats were identified by passing the assembly first through the REPET package (v2.2). The TEdenovo pipeline (Flutre *et al.*, 2011) was used to build a library of consensus sequences representative of repetitive elements in the genome assembly. The library of consensus sequences was classified using PASTEC classifier followed by semi-manual curation. A library of manually curated TEs from other diatoms was appended to the TEdenovo library and

redundancy was removed from the combined library. The TEannot pipeline (Quesneville *et al.*, 2005) was then launched with default settings using the sequences from the filtered combined library as probes to perform genome annotation.

Full length complete LTRs in the *P. multistriata, P. multiseries, F. cylindrus, P. tricornutum, T. pseudonana* and *O. sativa* genomes were identified using the LTRHarvest (parameters: -similar 50) and LTRDigest (parameters: -pdomevalcutoff 0.000001) which are part of the Genome-tools package v1.5.4 (Gremme *et al.*, 2013). The LTRHarvest predictions were filtered by LTRDigest based on presence of at least one LTR associated protein domain (reverse transcriptase, RNAseH, integrase, protease, gag and env) using HMMER3 package v3.1b1 (Eddy, 2011). The LTR associated protein domain HMM profiles were extracted from PfamA database. The Pfam IDs were obtained from previously published studies (Wang & Liu, 2008; Grau *et al.*, 2014). The relative age of LTR insertion was estimated using the method proposed in previous studies (Kimura, 1980). During the LTR retrotransposon replication cycle the two LTRs of a new insert are identical in sequence but they accumulate mutations to diverge over time. An insertion date can be estimated using the calculated divergence and a general substitution rate, with the equation: $T = D/2t$, where T is the time elapsed since the insertion, D the estimated LTR divergence and t the substitution rate per site per year. A substitution rate of $7.5*10^{-9}$ substitutions per site per year is proposed by Sorhannus and Fox for diatom nuclear genes (Sorhannus & Fox, 1999) while Gaut proposed a substitution rate of $6.5*10^{-9}$ substitutions per site per year for nuclear genes in grasses (Gaut *et al.*, 1996). However Ma and Bennetzen estimated this rate to be twice for LTRs in grasses ($1.3*10^{-8}$)(Ma & Bennetzen, 2004). Hence the substitution rate for LTRs in diatoms was taken as twice that of nuclear genes ($1.5*10^{-8}$). The inverted repeats in a complete copy were aligned with ClustalW v2.0.10 and the divergence was calculated using the baseml binary from PAML package v4.8 with the Kimura 2 parameter model.

Comparison of gene builds in different organisms

Gene annotations for the following genomes were downloaded in GFF3 format along with genome sequence in FASTA format

- *P. tricornutum* (http://genome.jgi-psf.org/Phatr2/Phatr2.home.html)
- *T. pseudonana* (http://genome.jgi-psf.org/Thaps3/Thaps3.home.html)
- *T. oceanica* (http://protists.ensembl.org/Thalassiosira_oceanica_ccmp1005/Info/Annotation/)
- *P. multiseries* (http://genome.jgi.doe.gov/Psemu1/Psemu1.home.html)
- *F. cylindrus* (http://genome.jgi.doe.gov/Fracy1/Fracy1.home.html)
- *P. ultimum* (http://pythium.plantbiology.msu.edu/download.shtml)
- *E. siliculosus* (https://bioinformatics.psb.ugent.be/gdb/ectocarpus/)

The statistics for the genomic features were extracted from the GFF files using custom shell script and the BEDTools package v2.22 binaries (substractBed, mergeBed, groupBy). The genome size, N50 value and GC content were taken from the respective publications (Armbrust *et al.*, 2004; Bowler *et al.*, 2008; Cock *et al.*, 2010; Lévesque *et al.*, 2010; Lommer *et al.*, 2012; Tanaka *et al.*, 2015; Mock *et al.*, 2017) or, if not mentioned, calculated from the genome sequence using stats.sh script from BBTools v36.92 (http://jgi.doe.gov/data-and-tools/bbtools/).

Identification of conserved non-coding elements

Genome sequences (repeats masked in lowercase), gene models (GFF3 format) and ESTs (fasta) were downloaded for the following species in FASTA format.

- *P. multiseries* (http://genome.jgi.doe.gov/Psemu1/Psemu1.home.html)
- *F. cylindrus* (http://genome.jgi-psf.org/Fracy1/Fracy1.home.html)
- *P. tricornutum* (http://genome.jgi-psf.org/Phatr2/Phatr2.home.html)
- *T. pseudonana* (http://genome.jgi-psf.org/Thaps3/Thaps3.home.html)

The EST sequences were mapped to their respective genomes with BLASTn v2.2.30 (Camacho *et al.*, 2009) and parsed into BED format with custom Perl scripts using BioPerl libraries. The genome sequences in FASTA format were aligned pairwise against the reference *P. multistriata* v1.4 genome with LASTZ v1.02.00 (--ambiguous=iupac --strand=both --format=maf H=2000 Y=3400 L=6000 K=2200). Utilities from the UCSC source code tree were used to generate NET alignments from the raw pairwise alignments. The following steps were performed

- Convert pairwise alignment from MAF to AXT format (axtChain).

- Build chains from AXT files (axtChain -linearGap=loose).
- Convert chains into AXT format (chainToAxt, axtSort, axtToMaf)
- Build pre-chains (chainPreNet)
- Build net (chainSort, chainNet -minSpace=1, netSyntenic)
- Convert net to MAF format (netToAxt, axtSort, axtToMaf)

The pairwise NET alignments in MAF format were combined into a single diatom NET alignment file using the roast binary from the MULTIZ package v11.2 with *P. multistriata* as reference genome. Custom Perl scripts were used to scan the diatom NET alignment to identify conserved intergenic blocks (window 20 bp, step 10 bp) which do not overlap gene/EST features in the species conserved. The UCSC utility mafsInRegions was used to extract the alignment for each block and intersectBED from the BEDTools package v2.22 was used to check overlap against gene/ESTs. The conserved intergenic blocks are classified into multiple subsets based on the species where they lie conserved. Overlapping conserved intergenic blocks for each species subset were merged to obtain 13 subsets comprising of 2,296 conserved intergenic elements. The conserved regions with >= 50% N (unresolved sequences) were filtered to obtain 1,886 elements. The trnaScan-SE software was employed to identify tRNA genes in the *P. multistriata* genome. The conserved intergenic elements were searched for homology against UniprotKB database (UniProt Consortium, 2012), RFAM database, *P. multistriata* tRNAs and SILVA rRNA database using BLASTn/BLASTp v2.2.30 (default parameters) to filter potential contamination from partial proteins and non-coding RNAs. Post-filtering 1,564 conserved non-coding elements (CNEs) were identified in the *P. multistriata* genome. The relative distance of the CNEs from transcription start sites (ab-initio gene models) and random genomic sites excluding predicted TSS (shuffleBed -excl -noOverlapping) was calculated with “bedtools reldist” from the BEDTools v2.22 package. The closest TSS for each CNE was obtained by the closestBed binary (-t "first") from the BEDTools v2.22 package. The gene ontology enrichment analysis (on genes represented by the closest TSS) was performed on the GO mapping generated by the Annocript pipeline using an R script from Annocript_utils repository (https://github.com/frankMusacchia/Annocript_utils). The script uses Fisher exact test and p-value FDR correction to select significantly enriched GO classes in the given subset of genes

compared to total number of genes (minimum representatives for a GO class: 5 genes; FDR <= 0.05). Random genomic regions (size matched to CNEs), excluding locations conserved with other diatoms were obtained using shuffleBed (-excl -noOverlapping) binary from the BEDTools package v2.22. The CNE and the shuffled sequences were scanned for transcription factor binding sites using the JASPAR 2014 database and custom Perl scripts using the Perl TFBS modules at >= 80% binding threshold. A hypergeometric test was performed to determine the transcription factor families with a significant frequency of binding sites in CNEs with respect to shuffled genomic regions using the R dhyper function (p-value adjusted <= 0.05). The Pfam accessions for transcription factor families which show an enrichment of binding sites on the CNEs were obtained from ID mapping reported in a previous publication (Todd *et al.*, 2014) and the Plant transcription factor database (Jin *et al.*, 2014). The HMM profiles for the enriched transcription factor families were extracted from the PfamA HMM database and compared against peptide sequences of *P. multistriata* gene models using the hmmsearch (--max -E 0.001 --domE 0.001 --incE 0.001 --incdomE 0.001) binary from HMMER3 package v3.1b1.

Expansion of gene families in *P. multistriata*

Proteomes of Stramenopiles (*P. multistriata, P. multiseries, F. cylindrus, P. tricornutum, T. pseudonana, A. candida, P. sojae, P. ultimum, S. parasitica, E. siliculosus, A. anophagefferens, N. gaditana, B. hominis*) were compared against profile HMMs of protein families classified in the SUPERFAMILY database (Wilson *et al.*, 2009). The comparison was performed using Perl scripts provided by the SUPERFAMILY database (http://supfam.cs.bris.ac.uk/SUPERFAMILY/howto_use_models.html) and the hmmscan binary from HMMER3 software (default e-value 0.01). For each SUPERFAMILY present in *P. multistriata* a Z-score was calculated using the following formula (no. of SUPERFAMILY genes in *P. multistriata* – mean no. of SUPERFAMILY genes in all proteomes) / standard deviation of SUPERFAMILY genes in all proteomes. Further, Z-score values for each Superfamily were obtained using the following criteria

- *P. multistriata* against all other proteomes.
- *P. multistriata* against all other proteomes except *P. multiseries.*

- *P. multistriata* against all other proteomes except *P. multiseries, F. cylindrus.*
- *P. multistriata* against all other proteomes except other diatoms.

If any Z-score value is above 2 with at least 5 members in *P. multistriata* for a given SUPERFAMILY, it is considered to be expanded and further sub-classified into species-specific or diatom-specific expansion event.

Identification of potential gene families by clustering of protein sequences

Complete bacterial (1,116 species) and archaeal (121 species) proteomes were obtained from OrthoDB v7 (Waterhouse *et al.*, 2013) and arCOG (Wolf *et al.*, 2012) databases in FASTA format. The CD-HIT software was used to further remove the redundancy within the databases at default parameters. The bacterial proteomes were separated into subsets based on their phyla before clustering by CD-HIT, except for the phylum proteobacteria which was further sub-classified into major classes (Supporting Information Table S6). Further, 50 eukaryotic proteomes (from sequenced genomes) broadly representing the tree of life were downloaded in FASTA format from Uniprot (http://www.uniprot.org/help/softwarematic_access) and clustered with CD-HIT at default parameters except when:

- The proteome is unavailable in Uniprot (*B. natans*, *P. multiseries, F. cylindrus, S. minutum*).
- The number of proteins reported in Uniprot is significantly higher than the number of genes in an organism thus rendering the CD-HIT clustering step inefficient (*H. sapiens, D. melanogaster, M. leidyi, S. moellendorfii, A. thaliana, O. sativa*).

In such cases the proteome is obtained from Ensembl or from the respective genome database. Here the clustering step is skipped by considering only the longest protein sequence for each gene. The protein sequences from archaea, bacteria and eukaryota along with the *P. multistriata* proteome were combined to create a FASTA database. The FASTA headers were formatted to assign a numerical ID and taxonomic class to each sequence. The mapping of numerical IDs to their respective accession and description was stored in a MySQL database. An All vs All BLASTp search was performed on the combined FASTA file (-outfmt 6 -evalue 1e-5 -word_size 4 -threshold 18 -seg 'yes' -max_target_seqs 100000 -dbsize 2543962). The results of the BLASTp search were provided to the orthAgogue software (--overlap 50 --use_scores)

(Ekseth *et al.*, 2014) for estimation of homology between the protein sequences. Here it is important to note that the orthAgogue software employs a coverage cut-off of >= 50% along with consideration for the BLASTp score for all hsps of a given protein. Compared to the simpler best reciprocal BLAST hit approach, these measures add stringency to the prediction, which aid in defining the downstream tree topology for a given cluster. The “abc” format output from orthAgogue was given to the MCL software (--abc -I 1.5) (Enright *et al.*, 2002) for clustering of the proteins into homologous groups. The MCL output was processed by custom shell script to assign a group ID to each predicted cluster of homologous proteins. Venn diagrams for shared gene families and *P. multistriata* proteins sharing orthologs with other taxonomic groups were generated using the VennDiagram package in R (http://cran.r-project.org/web/packages/VennDiagram/index.html).

Estimation of gene family gains and losses in Stramenopiles

Clusters containing only one-to-one orthologs of each stramenopile species (85 clusters, considering 13 species mentioned in “Expansion of gene families in *P. multistriata*” section) were chosen to generate the species tree for Stramenopiles. In brief

- All stramenopile proteins in each one-to-one ortholog clusters were aligned using MAFFT v7.205 (--maxiterate 1000 –localpair).
- The alignments build were concatenated and trimmed with trimAl software v1.4 (-gappyout).
- ProtTest software v3.2 (Darriba *et al.*, 2011) was run on the trimmed concatenated alignment to figure out the best amino acid substitution matrix to generate a phylogenetic tree based on the Bayesian Information Criterion score (all-matrices -all-distributions -F).
- The model suggested by ProtTest was LG model with invariant sites plus gamma distribution along with empirical amino acid frequencies. Hence this model was used to generate the phylogenetic tree using a maximum likelihood as well as bayesian approach.

The maximum likelihood tree was generated with RaxML software v8.1.3 (-f a -m PROTGAMMAILGF -p 12345 -x 12345 -# 1000). Next, protein clusters with at least one member from any stramenopile species were identified to obtain 28,927 clusters. For each stramenopile species a binary code was established stating the presence of absence of the species in each of

the 28,927 clusters using a custom Perl script. The binary format file along with the maximum likelihood tree was subjected to a Dollo parsimony analysis using Dollo binary from the Phylip package v3.696. The results of Dollo binary was parsed using a Perl script (extract_dollop_output_sequences_v2-fast.pl) obtained from https://github.com/guyleonard/orthomcl_tools to identify gene family gain and loss events at each branch point of the tree. It is worth mentioning that genes lacking significant similarity with other species, yet clustered with another member of the same species are presented as a species-specific gene gain event, while those which remain as species-specific singletons were considered as singletons where the measure of similarity is at least 50% reciprocal coverage and an e-value less than 1E-5. Further to confirm the observation from the maximum likelihood tree a bayesian tree was inferred on the same alignment using the same substitution model with MrBayes v3.2.6 (prset aamodelpr=fixed(lg) statefreqpr=fixed(empirical); lset nst=6 rates=invgamma ngammacat=4; mcmc ngen=100000 samplefreq=250 printfreq=1000 nchains=8 temp=0.2 savebrlens=yes starttree=random; set seed=21343; sumt burnin=5000; sump burnin=5000). In both maximum likelihood and bayesian approaches *Blastocystis hominis* was considered as the outgroup. The topology of the maximum likelihood and bayesian inferred trees were compared with treedist binary from the Phylip software, which gave a symmetric distance of 0 and a branch score distance of 0.6 between the two trees. This indicates an identical tree topology inferred by both the approaches, thus adding support to the inferred species associations. The GO terms associated with Uniprot protein IDs for all stramenopile species considered (see “Expansion of gene families in *P. multistriata*” for species list) were obtained using the Uniprot REST service and custom Perl scripts except for *Pseudo-nitzschia* species and *F. cylindrus*. The species exempted lack Uniprot annotations hence the Annocript (Musacchia *et al.*, 2015) annotations for the given transcriptomes were used to extract the mapped GO terms. GO terms associated with all members from stramenopile species were assigned as a non-redundant set to each cluster considered in the gene family gain/loss analysis. The GO term enrichment analysis was performed by using Fisher exact test and p-value FDR correction to select significantly enriched GO classes in the given subset of

clusters compared to all clusters considered in the analysis (minimum representatives for a GO class: 5 clusters; FDR <= 0.05).

Identification of genes acquired from red algae and by horizontal gene transfer from bacteria in *P. multistriata*

Identification of horizontal gene transfer (HGT) events in *P. multistriata* was performed with the following steps:

- Identify protein clusters (generated in the previous section) which contain at least one *P. multistriata* protein and extract the sequences of the members.
- Build a multiple alignment for each cluster (with member protein sequences) using MAFFT software (--maxiterate 1000 –localpair)(Katoh & Standley, 2013). The generation of a phylogenetic tree is highly dependent on the accuracy of the multiple alignment, hence the MAFFT program was used to align the proteins at high sensitivity mode which is reported to significantly outperform other multiple alignment algorithms.
- Trim columns with >=95% gaps in the alignment generated using trimAl software (-gt 0.05) (Capella-Gutiérrez *et al.*, 2009).
- Generate a phylogenetic tree with the trimmed alignment using the FastTree software at high sensitivity (-gamma -mlacc 2 -slownni -slow -spr 4) using both JTT and WAG models. The tree generation and parsing was automated with custom Perl scripts using BioPerl tree parsing modules. The phylogenetic trees for each cluster were parsed to identify genes of potential bacterial origin using the following criteria:
- Find a clade of interest represented in majority by bacteria, archaea and diatoms (>= 90%) but without members of metazoa, plantae or fungi.
- Bootstrap cut-off at the clade of interest >= 0.5 or the average bootstrap value for the tree is >= 0.5. If one of the bootstrap values are <= 0.5 the tree is still retained (if other filters are passed) as a candidate with medium confidence.
- To add further stringency to the analysis at least 5 bacterial members must be present in the clade of interest (10 in case *P. multistriata* is the only eukaryote in the clade) to avoid false

positives due to misplacement of a single protein within clade of another taxa which can be caused from issues such as long branch attraction.

It is reported that the JTT and WAG substitution models are better suited for phylogeny inference in vertebrates and bacteria respectively (Keane *et al.*, 2006). Hence finally the steps mentioned above are repeated for the trees generated on the same cluster using JTT and WAG amino acid substitution models and, if both the trees pass the filtering criteria, the given *P. multistriata* protein in the clade of interest is predicted to be acquired by an HGT event from bacteria. The same criteria was used to identify genes of bacterial origin in Stramenopiles and SAR by considering >=90% of the clade comprising bacteria and/or archaea with Stramenopiles/SAR. The GO term enrichment analysis for genes acquired by HGT was performed by using Fisher exact test and p-value FDR correction to select significantly enriched GO classes in the given subset of genes compared to all genes in *P. multistriata* (minimum representatives for a GO class: 5 genes; FDR <= 0.05). The phylogenetic trees for each cluster were parsed to identify genes of potential red algal origin by searching for a clade of interest which has genes belonging to Stramenopiles and at least one rhodophyta without any metazoa, fungi or green plants.

Co-culture experiments

The bipartite glass apparatus used for co-culturing experiments (Paul *et al.*, 2012) consists of two glass bottles (Duran flasks: VWR, Dresden, Germany) each having ca. 500 ml holding capacity with flat edge opening of 100 mm in diameter that allows connecting two bottles (Fig. 4A). To fill and collect culture samples, an additional neck like opening was generated in each bottle. The glass bottles are held together by a holding clamp. A hydrophilic polyvinylidene fluoride (PVDF) membrane filter (Durapore, Millipore, Billerica, MA, US) with 0.22 µm pore size was placed in between the two bottles to keep the cells separate. An O-ring made up of silicon was placed above the filter, between the two bottles, to ensure leak-proof set up.

Two independent co-culturing experiments using four strains, MT+ B856 with MT- B939, and MT+ B938 with MT-B857, were performed to collect RNA samples for RNA-seq (Supporting

Information Fig. S10). An additional experiment was performed with the pair B937 (MT+) and B936 (MT-) for qPCR validations. Cell concentration was 80,000 cells/ml for each strain.

Synchronization of the cell cycle by prolonged dark incubation and flow cytometry analyses

Dark induced cell cycle synchronization was employed in order to get the maximum number of cells in one cell cycle phase so as to induce sexual phase synchronously. After 36 hours dark incubation, cultures were reilluminated and 50 ml samples were centrifuged and resuspended in cold methanol (100%) and stored at -20 ^{0}C until analysis. Successively, samples were resuspended in TE buffer, treated with RNase I (300 µg ml^{-1}) for 45 min and stained with SYBR Green (1:10000 dilution of SYBR® Green I - 10,000X concentrate, Invitrogen, Thermofisher, Waltham, MA, US) for 15 min. Samples were collected at two additional time points, 2 hours and 6 hours after light re-illumination. Cell cycle synchronization was verified with a FACSCalibur flow cytometer (Becton Dickinson BioSciences Inc., Franklin Lakes, NJ, US) with standard filters and a 488 nm Ar laser. SYBR Green fluorescence (DNA) was collected through 530 +/- 30 nm optical filters in order to assess the percentage of cells in the different cell cycle stages. Control cells presented always a bimodal distribution of SYBR Green fluorescence, allowing to assess cell cycle blockage (one peak) in treated samples. Sample acquisition was realized using the BD CellQuest software, while relative proportions of cells in the different stages of the cell cycle were assessed using the ModFit software (Verity Inc., Palo Alto, CA, US). In experiments in which cells were left in the co-culturing apparatus for a longer time period (up to 36 hours, a time at which gametes can generally be observed in plates containing both MT+ and MT- cells) the formation of gametes was never recorded, indicating that physical contact between the two MTs is required for meiosis to occur (data not shown).

Sample collection, RNA extraction and sequencing

To induce the sexual phase, synchronized cultures of opposite mating type were co-cultured in the bipartite glass apparatus. Simultaneously, MT+ and MT- strains were grown in separate glass flasks as controls (Fig. 4A). To verify success of reproduction, a control experiment was set up in a 6-well culture plate where MT+ and MT- strains were grown together allowing direct

physical contact. Production of gametes was observed in the control culture 24 hours after mixing MT+ and MT- strains together (data not shown).

Two and six hours after the co-culture started, samples for RNA extraction were collected from the two strains in co-culture and from the parental strains cultivated in monoculture. Samples were collected onto 1.2 µm pore-size membrane filters (RAWP04700 Millipore, Billerica, MA, US), placed in Trizol™, flash frozen in liquid nitrogen immediately and stored at -80 ^{0}C until RNA extractions. To test the synchronization of cultures two samples from monocultures (MT+ and MT-) were collected before starting the experiment.

RNA was extracted according to the manufacturer's instructions (Trizol reagent, Invitrogen, Thermofisher, Waltham, MA, US) and the genomic DNA contamination was removed by DNase I treatment (RNase-Free DNase Set Qiagen, Hilden, Germany) followed by RNA purification using RNeasy Plant Mini Kit (Qiagen, Hilden, Germany). The quantity of RNA was determined by Qubit assay (Qubit® 2.0 Fluorometer, Life Technologies, Thermofisher, Waltham, MA, US) and integrity was assessed by Bioanalyzer (2100 Bioanalyzer Instruments, Agilent Technologies, Santa Clara, CA, US) as well as by running samples on a 1.5% agarose gel.

Libraries were prepared using a Beckman Biomek FX laboratory automation workstation and the Illumina® TruSeq® Stranded Total RNA Sample Preparation kit (Illumina, San Diego, CA, US), following the standard procedure for poly-A selection and starting with 500 ng total RNA. Samples were sequenced on Illumina HiSeq2000 producing single end 50 bp reads. Library preparation and sequencing were done at the Genecore Facility of the EMBL, Germany.

RNA-seq filtering, mapping and differential expression analysis

The raw sequencing reads from all samples were processed with the Trimmomatic program (Bolger *et al.*, 2014) to trim low quality bases, filter reads with low quality and smaller than 36 bases. The quality control resulted in removal of 1-2% of reads from all the samples (parameters: ILLUMINACLIP::2:30:10 LEADING:3 TRAILING:3 SLIDINGWINDOW:4:15 MINLEN:36 HEADCROP:5). Further the STAR aligner program (Dobin *et al.*, 2013) was used to map the filtered reads on the *Pseudo-nitzschia multistriata* genome v1.4 (parameters: -outFilterMultimapNmax 5 --seedMultimapNmax 1000 --seedSearchStartLmax 25 --

outFilterMismatchNoverLmax 0.01 --outFilterMatchNminOverLread 0.9 --outFilterIntronMotifs RemoveNoncanonical --outSAMstrandField intronMotif --outSJfilterCountUniqueMin 5 5 5 5 --outFilterType BySJout).

The Augustus gene models were associated to the mapped reads from each sample to generate raw read counts for each gene as a measure of their expression level (multiBamCov -split). The samples obtained from 2 hours and 6 hours time points were analyzed separately. The edgeR program v3.6.1 (Robinson *et al.*, 2010) was used to obtain the differentially expressed genes in the T1 and T2 samples using three comparisons at each time point employing generalized linear models (FDR <= 0.05, log fold change >= +/-1)

- Control vs Sexualized for both MTs.
- Control vs Sexualized for MT+.
- Control vs Sexualized for MT-.

While in the control vs sexualized comparisons for both MTs, the state of the cell (control vs sexualized) and the MT (+, -) are taken as the factors for estimating dispersion, in case of the control vs sexualized comparison for each MT, the cellular state remains a factor along with the intra-strain variation. Mapped reads for the 16 libraries produced can be visualised as tracks on the genome browser, library codes displayed on the genome browser are given in Supporting Information Table S9.

qPCR validations

For validation of RNA-seq differential expression data, total RNA was extracted from samples collected at 6 h. One microgram of total RNA was reverse transcribed into cDNA using QuantiTect® Reverse Transcription Kit (Qiagen, Hilden, Germany) following manufacturer's instructions. cDNAs quality was tested under standard PCR conditions and PCR products were electrophoresed in 1.5% agarose gel. 1:10 dilutions of the cDNAs were used in qPCR amplification.

19 candidate genes were selected for gene expression validation by qPCR. Primers for the selected genes (Supporting Information Table S1) were designed manually using EditSeq software (DNASTAR Inc., Madison, WI, US). To find the best normalization gene, expression

levels of four reference genes, *COPA*, *CDK-A*, *ACT* and *TUB-A* (Adelfi *et al.*, 2014) and four target genes were investigated. After geNorm analysis (Vandesompele *et al.*, 2002), *TUB-A* was found to be the most stable gene across experimental conditions and was further used as a reference gene for normalization. Real time PCR amplification was performed using 1 µl of cDNA (1:10 dilution), 4 µl of the primers (final concentration 0.7 µM of each primer) and 5 µl of Fast SYBR Green Master mix with ROX (Applied Biosystems, Foster City, CA, US) in a final volume of 10 µl, using ViiA™ 7 Real-Time PCR System (Applied Biosystems, Foster City, CA, US). PCR conditions used were as follows: 95 °C for 20 s, 40 cycles at 95 °C for 1 s and 60 °C for 20 s, 95 °C for 15 s, 60 °C 1 min, and a gradient from 60 °C to 95 °C for 15 min. The results were analyzed and collected in Excel sheet using the ViiA™ 7 Software.

Expression analysis was performed using the Relative Expression Software Tool-Multiple Condition Solver (REST-MCS), the calculation software for the relative expression in qPCR, using Pair Wise Fixed Reallocation Randomization Test.

Identification of homologous genes and Ka/Ks analysis

The analyzed data included 12,152 and 19,703 CDS sequences of *P. multistriata* and *P. multiseries* (Psemu1, downloaded from JGI), respectively. As a first step, a reciprocal best BLAST hit (RBH) approach was used to identify *P. multistriata* and *P. multiseries* orthologous sequences. Only alignments covering at least 30% of *P. multistriata* sequences were retained. The RBH was calculated using both the e-value and the bit-score of the alignment and they produced the same results, identifying 7,128 *P. multistriata* and *P. multiseries* reciprocal best BLAST hits. As a following step each pair of sequences of *P. multistriata* and *P. multiseries* were aligned with Prank v.150803 (Löytynoja, 2014), using empirical codon model and the alignments were refined by using trimAl v1.4.rev15 (Capella-Gutiérrez *et al.*, 2009) to remove gaps and badly aligned regions. Of the 7,128 processed alignments, 6,066 (85%) were suitable for Ka/Ks calculation. Ka/Ks calculation was performed with KaKs_Calculator (Wang *et al.*, 2010), the model for the calculation was chosen for each alignment by using the AICc model selection method.

Data availability

The *Pseudo-nitzschia multistriata* assembly has been deposited at ENA under the accession PRJEB9419.

RNA-seq reads for the six samples used for the *de novo* transcriptome are available at http://genomeportal.jgi.doe.gov/pages/dynamicOrganismDownload.jsf?organism=PseNitnscriptome_FD and at http://genomeportal.jgi.doe.gov/pages/dynamicOrganismDownload.jsf?organism=Psenittraphasell.

RNA-seq reads for the 16 samples from the co-culturing experiments have accession E-MTAB-5469.

Gene models and annotation are accessible via Zenodo (doi: 10.5281/zenodo.495408).

The phylogenetic trees for all protein clusters containing a *P. multistriata* member and a MySQL database with protein ID correspondences are accessible via Zenodo (doi: 10.5281/zenodo.345101).

Information deriving from the analyses conducted on genomic and transcriptomic data produced in this work can be visualized in the *P. multistriata* genome browser: http://apollo.tgac.ac.uk/Pseudo-nitzschia_multistriata_V1_4_browser/sequences

Username and password are both "pnitzschia".

Tracks available in the genome browser:

Gene prediction- gene models tracks, track “Genes V3 WA” shows the gene models with annotation.

Conservation- pairwise alignment with other diatoms, psmu, *Pseudo-nitzschia multistriata*, psmus, *Pseudo-nitzschia multiseries*, frcy, *Fragilariopsis cylindrus*, phtr, *Phaeodactylum tricornutum*, thps, *Thalassiosira pseudonana*.

Repeats- Repeats found with different prediction programs.

RNA-Seq Data- RNA-seq reads, correspondence between numbers and samples can be found in Supporting Information Table S9.

Non Coding- CNEs, rRNAs and tRNAs predictions.

Homologous Protein Alignments- Homology with *P. tricornutum* and *T. pseudonana* proteins.

Software versions

Genome sequencing and assembly

NextClip v0.7

Allpaths-LG v44837

RAMPART v0.7.0

Gene prediction and annotation

Trinity r20131110

PASA v20130907

Augustus v2.7

Exonerate v2.2.0

Annocript v1.1.3

blastx v2.2.30

rpsBlast v2.2.29

dna2pep v1.1

Portrait v1.1

Repeat Annotation

REPET v2.2

PASTEC

LTRHarvest, genometools v1.5.4

LTRDigest, genometools v1.5.4

bedtools v2.22

Identification of conserved non-coding elements

Lastz v1.02

Multiz v11.2

trnaScan-SE v1.3.1

Expansion of gene families in P. multistriata

Hmmer v3.1

Identification of potential gene families by clustering of protein sequences

blastp v2.2.30

CD-HIT v4.6.1

Orthagogue v1.0.3

mcl v14-37

VennDiagram 1.6.9

Estimation of gene family gains and losses in Stramenopiles

mafft v7.205

trimal v1.4

prottest v3.2

RaxML v8.1.3

MrBayes 3.2.6

Phylip 3.696

Identification of genes acquired from red algae and by horizontal gene transfer from bacteria in P. multistriata

mafft v7.205

trimal v1.4

RNA-seq filtering, mapping and differential expression analysis

trimmomatic v0.32

STAR v2.3

edgeR v3.8.6

Identification of homologous genes and Ka/Ks analysis

Prank v.150803

trimal v1.4

KaKs_calculator

References

Adelfi MG, Borra M, Sanges R, Montresor M, Fontana A, Ferrante MI. **2014**. Selection and validation of reference genes for qPCR analysis in the pennate diatoms Pseudo-nitzschia multistriata and P. arenysensis. *Journal of Experimental Marine Biology and Ecology* **451**: 74–81.

Armbrust EV, Berges JA, Bowler C, Green BR, Martinez D, Putnam NH, Zhou S, Allen AE, Apt KE, Bechner M, *et al.* 2004. The genome of the diatom Thalassiosira pseudonana: ecology, evolution, and metabolism. *Science* **306**: 79–86.

Bolger AM, Lohse M, Usadel B. **2014**. Trimmomatic: a flexible trimmer for Illumina sequence data. *Bioinformatics* **30**: 2114–2120.

Bowler C, Allen AE, Badger JH, Grimwood J, Jabbari K, Kuo A, Maheswari U, Martens C, Maumus F, Otillar RP, *et al.* 2008. The Phaeodactylum genome reveals the evolutionary history of diatom genomes. *Nature* **456**: 239–244.

Camacho C, Coulouris G, Avagyan V, Ma N, Papadopoulos J, Bealer K, Madden TL. **2009**. BLAST+: architecture and applications. *BMC Bioinformatics* **10**: 421.

Capella-Gutiérrez S, Silla-Martínez JM, Gabaldón T. **2009**. trimAl: a tool for automated alignment trimming in large-scale phylogenetic analyses. *Bioinformatics* **25**: 1972–1973.

Cock JM, Sterck L, Rouzé P, Scornet D, Allen AE, Amoutzias G, Anthouard V, Artiguenave F, Aury J-M, Badger JH, *et al.* 2010. The Ectocarpus genome and the independent evolution of multicellularity in brown algae. *Nature* **465**: 617–621.

Darriba D, Taboada GL, Doallo R, Posada D. **2011**. ProtTest 3: fast selection of best-fit models of protein evolution. *Bioinformatics* **27**: 1164–1165.

Dobin A, Davis CA, Schlesinger F, Drenkow J, Zaleski C, Jha S, Batut P, Chaisson M, Gingeras TR. **2013**. STAR: ultrafast universal RNA-seq aligner. *Bioinformatics* **29**: 15–21.

Eddy SR. **2011**. Accelerated Profile HMM Searches. *PLoS computational biology* **7**: e1002195.

Ekseth OK, Kuiper M, Mironov V. **2014**. orthAgogue: an agile tool for the rapid prediction of orthology relations. *Bioinformatics* **30**: 734–736.

Enright AJ, Van Dongen S, Ouzounis CA. **2002**. An efficient algorithm for large-scale detection of protein families. *Nucleic Acids Research* **30**: 1575–1584.

Flutre T, Duprat E, Feuillet C, Quesneville H. **2011**. Considering transposable element diversification in de novo annotation approaches. *PloS One* **6**: e16526.

Gaut BS, Morton BR, McCaig BC, Clegg MT. **1996**. Substitution rate comparisons between grasses and palms: synonymous rate differences at the nuclear gene Adh parallel rate differences at the plastid gene rbcL. *Proceedings of the National Academy of Sciences of the United States of America* **93**: 10274–10279.

Grabherr MG, Haas BJ, Yassour M, Levin JZ, Thompson DA, Amit I, Adiconis X, Fan L, Raychowdhury R, Zeng Q, *et al.* 2011. Full-length transcriptome assembly from RNA-Seq data without a reference genome. *Nature Biotechnology* **29**: 644–652.

Grau JH, Poustka AJ, Meixner M, Plötner J. **2014**. LTR retroelements are intrinsic components of transcriptional networks in frogs. *BMC Genomics* **15:** 626.

Gremme G, Steinbiss S, Kurtz S. **2013**. GenomeTools: A Comprehensive Software Library for Efficient Processing of Structured Genome Annotations. *IEEE/ACM Transactions on Computational Biology and Bioinformatics* **10**: 645–656.

Haas BJ, Delcher AL, Mount SM, Wortman JR, Smith RK, Hannick LI, Maiti R, Ronning CM, Rusch DB, Town CD, *et al.* **2003**. Improving the Arabidopsis genome annotation using maximal transcript alignment assemblies. *Nucleic Acids Research* **31**: 5654–5666.

Jin J, Zhang H, Kong L, Gao G, Luo J. **2014**. PlantTFDB 3.0: a portal for the functional and evolutionary study of plant transcription factors. *Nucleic Acids Research* **42**: D1182–D1187.

Katoh K, Standley DM. **2013**. MAFFT multiple sequence alignment software version 7: improvements in performance and usability. *Molecular Biology and Evolution* **30**: 772–780.

Keane TM, Creevey CJ, Pentony MM, Naughton TJ, Mclnerney JO. **2006**. Assessment of methods for amino acid matrix selection and their use on empirical data shows that ad hoc assumptions for choice of matrix are not justified. *BMC Evolutionary Biology* **6**: 29.

Kimura M. **1980**. A simple method for estimating evolutionary rates of base substitutions through comparative studies of nucleotide sequences. *Journal of Molecular Evolution* **16**: 111–120.

Lévesque CA, Brouwer H, Cano L, Hamilton JP, Holt C, Huitema E, Raffaele S, Robideau GP, Thines M, Win J, *et al.* **2010**. Genome sequence of the necrotrophic plant pathogen Pythium ultimum reveals original pathogenicity mechanisms and effector repertoire. *Genome Biology* **11**: R73.

Li H, Durbin R. **2009**. Fast and accurate short read alignment with Burrows-Wheeler transform. *Bioinformatics* **25**: 1754–1760.

Li H, Handsaker B, Wysoker A, Fennell T, Ruan J, Homer N, Marth G, Abecasis G, Durbin R, 1000 Genome Project Data Processing Subgroup. **2009**. The Sequence Alignment/Map format and SAMtools. *Bioinformatics* **25**: 2078–2079.

Lommer M, Specht M, Roy A-S, Kraemer L, Andreson R, Gutowska MA, Wolf J, Bergner SV, Schilhabel MB, Klostermeier UC, *et al.* **2012**. Genome and low-iron response of an oceanic diatom adapted to chronic iron limitation. *Genome biology* **13**: R66.

Löytynoja A. **2014**. Phylogeny-aware alignment with PRANK. *Methods in Molecular Biology* **1079**: 155–170.

Ma J, Bennetzen JL. **2004**. Rapid recent growth and divergence of rice nuclear genomes. *Proceedings of the National Academy of Sciences of the United States of America* **101**: 12404–12410.

Mock T, Otillar RP, Strauss J, McMullan M, Paajanen P, Schmutz J, Salamov A, Sanges R, Toseland A, Ward BJ, *et al.* **2017**. Evolutionary genomics of the cold-adapted diatom *Fragilariopsis cylindrus*. *Nature* **541:** 536–540.

Musacchia F, Basu S, Petrosino G, Salvemini M, Sanges R. **2015**. Annocript: a flexible pipeline for the annotation of transcriptomes able to identify putative long noncoding RNAs. *Bioinformatics* **31**: 2199–2201.

Paul C, Mausz MA, Pohnert G. **2012**. A co-culturing/metabolomics approach to investigate chemically mediated interactions of planktonic organisms reveals influence of bacteria on diatom metabolism. *Metabolomics* **9**: 349–359.

Quesneville H, Bergman CM, Andrieu O, Autard D, Nouaud D, Ashburner M, Anxolabehere D. **2005**. Combined evidence annotation of transposable elements in genome sequences. *PLoS computational biology* **1**: 166–175.

Quinlan AR, Hall IM. **2010**. BEDTools: a flexible suite of utilities for comparing genomic features. *Bioinformatics* **26**: 841–842.

Robinson MD, McCarthy DJ, Smyth GK. **2010**. edgeR: a Bioconductor package for differential expression analysis of digital gene expression data. *Bioinformatics* **26**: 139–140.

Sabatino V, Russo MT, Patil S, d'Ippolito G, Fontana A, Ferrante MI. **2015**. Establishment of Genetic Transformation in the Sexually Reproducing Diatoms *Pseudo-nitzschia multistriata* and *Pseudo-nitzschia arenysensis* and Inheritance of the Transgene. *Marine Biotechnology* **17**: 1–11.

Slater GSC, Birney E. **2005**. Automated generation of heuristics for biological sequence comparison. *BMC bioinformatics* **6**: 31.

Sorhannus U, Fox M. **1999**. Synonymous and Nonsynonymous Substitution Rates in Diatoms: A Comparison Between Chloroplast and Nuclear Genes. *Journal of Molecular Evolution* **48**: 209–212.

Stanke M, Schöffmann O, Morgenstern B, Waack S. **2006**. Gene prediction in eukaryotes with a generalized hidden Markov model that uses hints from external sources. *BMC Bioinformatics* **7**: 62.

Tanaka T, Maeda Y, Veluchamy A, Tanaka M, Abida H, Maréchal E, Bowler C, Muto M, Sunaga Y, Tanaka M, *et al.* **2015**. Oil Accumulation by the Oleaginous Diatom Fistulifera solaris as Revealed by the Genome and Transcriptome. *The Plant Cell* **27**: 162–76.

Todd RB, Zhou M, Ohm RA, Leeggangers HA, Visser L, Vries RP de. **2014**. Prevalence of transcription factors in ascomycete and basidiomycete fungi. *BMC Genomics* **15**: 214.

UniProt Consortium. **2012**. Reorganizing the protein space at the Universal Protein Resource (UniProt). *Nucleic Acids Research* **40**: D71-75.

Vandesompele J, De Preter K, Pattyn F, Poppe B, Van Roy N, De Paepe A, Speleman F. **2002**. Accurate normalization of real-time quantitative RT-PCR data by geometric averaging of multiple internal control genes. *Genome biology* **3**.

Wang H, Liu J-S. **2008**. LTR retrotransposon landscape in Medicago truncatula: more rapid removal than in rice. *BMC Genomics* **9**: 382.

Wang D, Zhang Y, Zhang Z, Zhu J, Yu J. **2010**. KaKs_Calculator 2.0: a toolkit incorporating gamma-series methods and sliding window strategies. *Genomics, Proteomics & Bioinformatics* **8**: 77–80.

Waterhouse RM, Tegenfeldt F, Li J, Zdobnov EM, Kriventseva EV. **2013**. OrthoDB: a hierarchical catalog of animal, fungal and bacterial orthologs. *Nucleic Acids Research* **41**: D358-365.

Wilson D, Pethica R, Zhou Y, Talbot C, Vogel C, Madera M, Chothia C, Gough J. **2009**. SUPERFAMILY--sophisticated comparative genomics, data mining, visualization and phylogeny. *Nucleic Acids Research* **37**: D380-386.

Wolf YI, Makarova KS, Yutin N, Koonin EV. **2012**. Updated clusters of orthologous genes for Archaea: a complex ancestor of the Archaea and the byways of horizontal gene transfer. *Biology Direct* **7**: 46.
